# Early Prediction of Multiple Organ Dysfunction in the Pediatric Intensive Care Unit

**DOI:** 10.3389/fped.2021.711104

**Published:** 2021-08-16

**Authors:** Sanjukta N. Bose, Joseph L. Greenstein, James C. Fackler, Sridevi V. Sarma, Raimond L. Winslow, Melania M. Bembea

**Affiliations:** ^1^Institute for Computational Medicine, The Johns Hopkins University, Baltimore, MD, United States; ^2^Department of Electrical and Computer Engineering, The Johns Hopkins University, Baltimore, MD, United States; ^3^Department of Anesthesiology and Critical Care Medicine, The Johns Hopkins University School of Medicine, Baltimore, MD, United States; ^4^Department of Biomedical Engineering, The Johns Hopkins University, Baltimore, MD, United States

**Keywords:** multiple organ dysfunction, organ failure, machine learning, early prediction, Pediatrics, critical care

## Abstract

**Objective:** The objective of the study is to build models for early prediction of risk for developing multiple organ dysfunction (MOD) in pediatric intensive care unit (PICU) patients.

**Design:** The design of the study is a retrospective observational cohort study.

**Setting:** The setting of the study is at a single academic PICU at the Johns Hopkins Hospital, Baltimore, MD.

**Patients:** The patients included in the study were <18 years of age admitted to the PICU between July 2014 and October 2015.

**Measurements and main results:** Organ dysfunction labels were generated every minute from preceding 24-h time windows using the International Pediatric Sepsis Consensus Conference (IPSCC) and Proulx et al. MOD criteria. Early MOD prediction models were built using four machine learning methods: random forest, XGBoost, GLMBoost, and Lasso-GLM. An optimal threshold learned from training data was used to detect high-risk alert events (HRAs). The early prediction models from all methods achieved an area under the receiver operating characteristics curve ≥0.91 for both IPSCC and Proulx criteria. The best performance in terms of maximum F1-score was achieved with random forest (sensitivity: 0.72, positive predictive value: 0.70, F1-score: 0.71) and XGBoost (sensitivity: 0.8, positive predictive value: 0.81, F1-score: 0.81) for IPSCC and Proulx criteria, respectively. The median early warning time was 22.7 h for random forest and 37 h for XGBoost models for IPSCC and Proulx criteria, respectively. Applying spectral clustering on risk-score trajectories over 24 h following early warning provided a high-risk group with ≥0.93 positive predictive value.

**Conclusions:** Early predictions from risk-based patient monitoring could provide more than 22 h of lead time for MOD onset, with ≥0.93 positive predictive value for a high-risk group identified pre-MOD.

## INTRODUCTION

Pediatric multiple organ dysfunction syndrome (MODS) occurs in more than 25% of children admitted to the pediatric intensive care unit (PICU) (1–3) and is one of the leading pathways to mortality in critically ill children (4). MOD has been defined as the presence of two or more concurrent organ dysfunctions (4–7). Multiple underlying conditions could lead to MOD, although sepsis stands out as the most commonly associated condition (3, 8–10) followed by trauma, burns, pancreatitis, inborn errors in metabolism, transplantation, and others (4). The all-cause incidence of MOD ranges between 6% in 283 admissions from a single PICU and as high as 57% in 3,671 admissions across nine PICUs (1, 2, 4, 9, 11–14). A higher number of dysfunctional organ systems is associated with increased risk of mortality regardless of the study population (8, 15, 16) with more than 75% difference in mortality between patients with the lowest and highest numbers of organ failures (9, 10). In a prospective study of 831 patients in a single PICU, more than 90% of deaths were associated with MODS (5, 6). Of children admitted to the PICU, 14–30% encounters have MOD on PICU day 1 (1, 14, 17) consisting of the largest proportion (78%) of all PICU MOD cases (18). New and progressive MOD (NPMOD) is defined as concomitant dysfunction of two or more organ systems occurring after PICU admission with no or single organ dysfunction, or additional dysfunctional organs following admission with MOD (19). NPMOD has been proposed as primary or secondary outcome instead of mortality in several pediatric critical care studies as PICU mortality has declined significantly over the last decades (19–22). There continues to be insufficient knowledge of the physiologic trajectory of patients who develop new, progressive, or persistent multiple organ dysfunction. We hypothesized that a complex set of physiologic patterns likely preceded adverse events such as simultaneous failure of more than one organ system.

Due to the inherent diversity in the cause and sequence of physiologic events that lead to MOD, the need to understand the risk factors for, and progression of, the syndrome calls for investigating ways to predict MOD, with the ultimate goal of optimizing monitoring, staffing, and early intervention regimens based on the progression of the underlying condition. The primary objective of this study was to develop data-driven early prediction models for identifying patients with increased risk of transitioning from no-MOD to MOD state. The secondary objective of this study was to explore if the time-evolving risk of developing MOD among positive predictions presented any clustering patterns and, if so, whether these patterns could be used as a risk-stratification method.

## Materials and Methods

### Study Design and Patients

We conducted a retrospective cohort study including all children <18 years admitted to the Johns Hopkins PICU between July 2014 and October 2015. This dataset included time series physiologic monitoring data from bedside monitors as well as electronic medical record data including ICD-9/10 diagnostic and procedure codes, vital signs, drug infusions, respiratory support, laboratory results, fluid input–output, and transfusion data. We excluded PICU encounters with missing hospital admission and discharge times, missing demographics, or vital signs, those with <2 h of PICU data preceding time of death and those with PICU day 1 MOD without recurrent transitions from no-MOD to MOD state. We further excluded no-MOD to MOD transitions with <15 min of data available prior to MOD transition. This study was approved by the Johns Hopkins Institutional Review Board (IRB00210572).

### Multiple Organ Dysfunction Definitions and Multiple Organ Dysfunction Label Evaluation

MOD is defined by the simultaneous presence of two or more organ dysfunctions (5–7, 18, 23). For this study, we used two widely accepted sets of organ dysfunction criteria published by Proulx et al. (4, 18) and International Pediatric Sepsis Consensus Conference (IPSCC) (7). We assigned and updated organ dysfunction labels every minute using the preceding 24-h windows, starting at 24 h from the time of PICU admission until PICU discharge. Organ dysfunction was evaluated starting 24 h from the time of PICU admission because no data was captured prior to PICU admission and reporting delay in laboratory test results might cause the first few hours to be labeled as not having organ dysfunction due to non-availability of measured data. Therefore, PICU encounters where MOD occurred within 24 h after admission and never recurred during the PICU course and those with <15 min of data prior to transition from a no-MOD to MOD state any time during the PICU course were excluded as data available prior to MOD transition was deemed insufficient.

We generated continuous binary MOD labels based on the presence of two or more simultaneous organ dysfunction as defined by the Goldstein (7) and Proulx criteria (4, 18). In order to populate infrequently measured physiologic data for continuous MOD labeling, we used carry-forward interpolation for a maximum period of 24 h (allowing up to 30 h only if a new test was ordered within 24–30 h of the previous) for laboratory tests and 6 h for vasoactive drug administration and continuous renal replacement therapy. We did carry forward interpolation for vital signs like non-invasive blood pressure, temperature, etc., which were not measured every minute. We defined *MOD transition* as the event when a patient transitioned from no organ or single-organ dysfunction to a state of at least two concomitant organ dysfunctions. We defined the interval of time over which the number of organ dysfunction remained the same as the *dwell time* in that state.

### Features

A total of 228 features extracted from minute-to-minute bedside monitor data and electronic medical record data were used to train predictive models. A missingness indicator variable was created for each infrequently measured value that is not routinely collected from all patients (see Section 1.1 in the Supplementary Materials and Supplementary Table 1).

### Model Building and Evaluation

The primary objective of this study was to build prognostic models, which could learn to distinguish physiologic patterns in the data collected prior to the development of MOD from those collected from non-MOD patients and provide a quantitative sense of likelihood of an impending no-MOD to MOD transition. We used different machine learning methods to build models that continuously output probability of developing MOD on a scale of 0–1, which we refer to as *risk score* in this study. The risk scores were also filtered by taking the median over the preceding 5 min windows to avoid sudden abrupt changes in risk scores from any outliers in minute-to-minute captured features. Using the time-varying risk scores evaluated on data collected prior to MOD transition as well as non-MOD patient data, we learned an optimal threshold value using the receiver operating characteristic (ROC) curve for each model. Since several criteria to choose thresholds from ROC curves have been described, we compared our model performances based on two different methods: point nearest from (0, 1) on ROC curve and point corresponding to maximum F1-score. We defined a *high-risk alert event* (HRA) as an instance when the risk score exceeded the optimal threshold and then remained above the threshold for at least 15 min. We defined the entire interval over which risk score stayed above the threshold as the *high-risk interval* (HRI), and the lead time between HRA and MOD transition as the *early warning time* (EWT).

We divided the complete set of eligible targets and controls into training and test sets by randomly splitting them in a 4:1 ratio in a stratified manner to preserve the relative ratio of MOD to non-MOD PICU admissions so that all MOD transitions from the same patient during a PICU stay are in the same data partition. We built predictive models for MOD transition events using training data consisting of up to 24 h of data collected prior to each MOD transition (target) and an equal number of randomly selected samples from no-MOD admissions (control). To test the model performance, we used the entire length of available data from both target and control periods to evaluate time-varying probability of developing MOD. The training set was subjected to 10-fold cross validation for parameter tuning and training performance assessment. We compared our model performance in terms of sensitivity, specificity, AUROC (area under ROC curve), accuracy, F1-score, PPV (positive predictive value), and NPV (negative predictive value) (24) (see Supplementary Section 1.2 on performance metrics). We also presented precision-recall curves in addition to ROC curves to account for the large class imbalance in the dataset. We compared the distribution of EWT, EWT normalized by length of available time and HRI across all positive predictions and reported if any difference in the distribution of these entities was observed across true positives and false positives using Wilcoxon rank sum test.

We compared the performance of models built using the following four methods: extreme gradient boosting (XGBoost) (25, 26), random forest-based probability machines (random forest) (27, 28), gradient boosted generalized linear models (GLMBoost) (29–31), and L1-regularized generalized linear models (LassoGLM) (24). All models were built in R (version 3.5.3) using the open source CRAN packages: xgboost (26), ranger (27), mboost (32), and glmnet (24), respectively, for the above methods. The choice of the above four methods was driven by the amount of available data, range of model complexity, and their capability of pruning features to yield a parsimonious model. The entire analysis, including model building, threshold selection, performance evaluation, and feature importance computation, was carried out independently for each of the above four applied methods.

Apart from being able to detect HRA events and evaluate EWTs in MOD transition cases, we also performed spectral clustering (33) on the risk score trajectories post-HRA to explore if the data resolved into distinct patterns and whether the accuracy of our predictions differ across different clusters. The spectral clustering method used in our analysis determined the number of clusters using the largest eigengap metric in the graph spectrum of similarity matrix built based on the K-nearest neighbor algorithm between each pair of positive predicted risk score trajectories in a 24-h window post-HRA. We also reported the positive predictive values across different risk groups by dividing the positive predicted cases into quartiles of risk scores at HRA, similar to analysis performed across deciles of risk scores by Liu et al. (34). Wilcoxon rank sum tests were used to compare the distribution of continuous variables such as EWT, HRI, etc., among different groups.

The choice of machine learning methods, training-test splitting, model building and threshold determination, performance metrics, and use of clustering and quartiles of risk-score at HRA to explore the presence of any underlying patterns in the risk score trajectories post-HRA were planned *a priori*. The observations made about differences in the distribution of EWT, HRIs (both unnormalized and normalized by length of available data) were based on inspection of the results obtained with our planned analysis.

### Feature Importance Evaluation

Global feature importance was computed on XGBoost and random forest model using the average fractional contribution of each feature in the total information gain or reduction in Gini index, respectively (using built-in functions in the XGBoost and ranger CRAN packages). For GLMBoost and LassoGLM, their risk scores are computed in the same manner as ordinary generalized linear models, and hence, the trained set of coefficients for each feature reflected its relative importance within a model. Since all of these four methods use different metrics to define variable importance, we reported the quantitative importance scores as relative importance on a scale of 0–100 such that the feature with the highest importance has a relative importance equal to 100. These feature importance metrics may not be accounted for perfectly if a variable is used more often in a model in lieu of a different feature that is highly correlated with it. For GLM (generalized linear model)-based models, relative importance was calculated as the absolute value of model coefficient for a feature divided by the highest absolute value of model coefficient across all features.

## Results

### Study Population Characteristics

**Figure 1:**
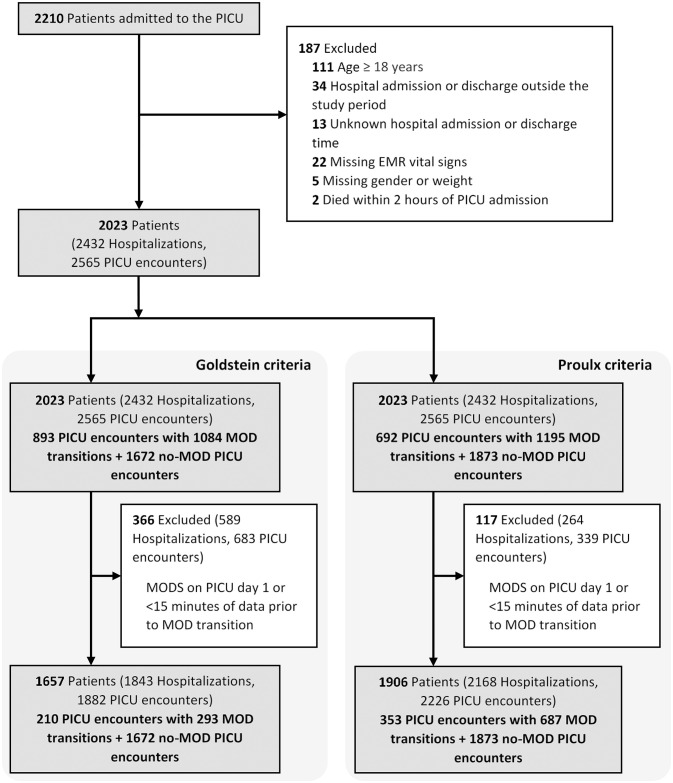
Study flowchart.

The final cohort included 2,023 patients with 2,565 PICU encounters (Figure 1 and Table 1). MOD was present in 893 (36.2%) and 692 (28.1%) of PICU encounters, using the IPSCC and Proulx criteria, respectively. Most MOD was present on PICU day 1, rendering a final data set of 293 and 687 MOD transitions using the IPSCC and Proulx criteria, respectively, that had >15 min of data prior to the time of transition. Rates of individual and multiple organ dysfunction are shown in Table 1. In bivariate analysis, age, weight, cardiac surgery during the admission, a diagnosis of status asthmaticus, pneumonia, sepsis, cardiac arrest, or renal insufficiency, chronic conditions (35, 36), and interventions including transfusions and ECMO, were significantly associated with MOD (p < 0.05) during the PICU encounter (Supplementary Table 2).

**Table 1:** Multiple and individual organ dysfunction rates using the International Pediatric Sepsis Consensus Conference (IPSCC) and Proulx et al. criteria of multiple organ dysfunction across 2,565 pediatric intensive care unit (PICU) encounters.

	**IPSCC criteria^**a**^**	**Proulx criteria^**a**^**	**Concordance^**b**^**
**MOD**
Multiple organ dysfunction (MOD) on PICU day 1	791 (30.84)	504 (19.65)	78.52%
Progressive MOD	146 (5.69)	68 (2.65)	94.39%
New and progressive MOD	893 (34.81)	692 (26.98)	77.97%
**Organ dysfunction**
Respiratory	1,165 (45.42)	1,515 (59.06)	72.71%
Neurological	955 (37.23)	274 (10.68)	73.45%
Cardiovascular	329 (12.83)	714 (27.84)	73.06%
Hepatic	347 (13.53)	103 (4.02)	86.82%
Hematological	279 (10.88)	198 (7.72)	92.32%
Renal	117 (4.56)	82 (3.2)	97.15%
Gastrointestinal^c^	–	19 (0.74)	–

a *Presented as counts (frequencies in %).*

b *Concordance measured as percentage of agreement between IPSCC and Proulx criteria.*

c *Gastrointestinal organ dysfunction is only defined in Proulx criteria and not in IPSCC criteria*.

### Continuous Organ Dysfunction Labels

**Figure 2:**
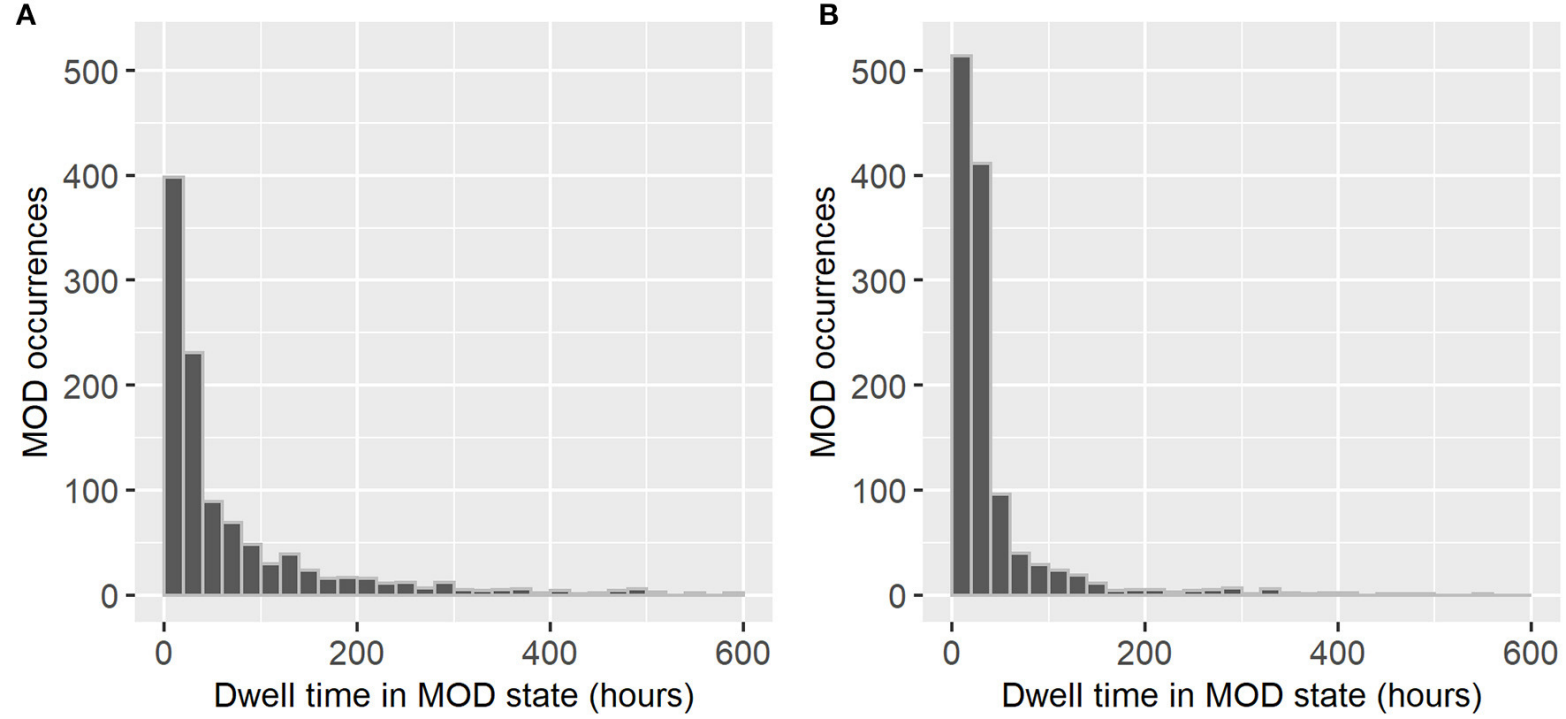
Dwell times in multiple organ dysfunction state using **(A)** International Pediatric Sepsis Consensus Conference (IPSCC) criteria and **(B)** Proulx et al. criteria for organ dysfunction.

Continuous evaluation of organ dysfunction status showed higher temporal instability in the Proulx criteria compared with the IPSCC criteria. The median (IQR, range) number of transitions in the number of organ dysfunctions across all PICU encounters with MOD was 3 (1–65) using the Proulx criteria vs. 2 (1–43) using the IPSCC criteria. The median (IQR, range) number of no-MOD to MOD transitions were 1 (11,0,1,2,3,4,5,6,7,8) for IPSCC criteria and 1 (1–16) for Proulx criteria. The median (IQR) dwell time in MOD state (at least two organ dysfunctions) was 24 (7.3–35.7) h using the Proulx criteria vs. 30 (9.6–90.1) h using the IPSCC criteria (Figure 2).

### Model Performance

**Figure 3:**
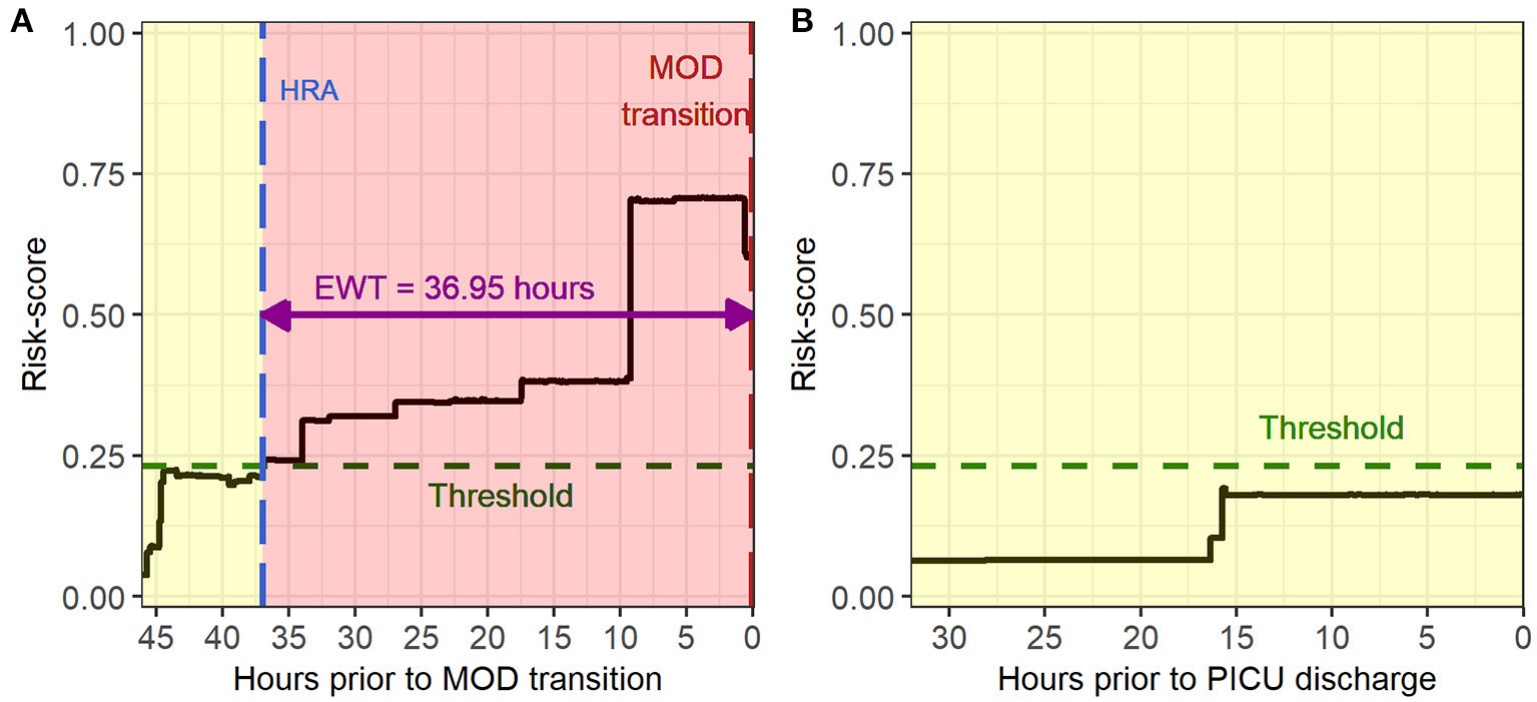
Example of time evolving risk scores in **(A)** a multiple organ dysfunction (MOD) transition case during a pediatric intensive care unit (PICU) encounter and **(B)** a no-MOD patient.

**Figure 4:**
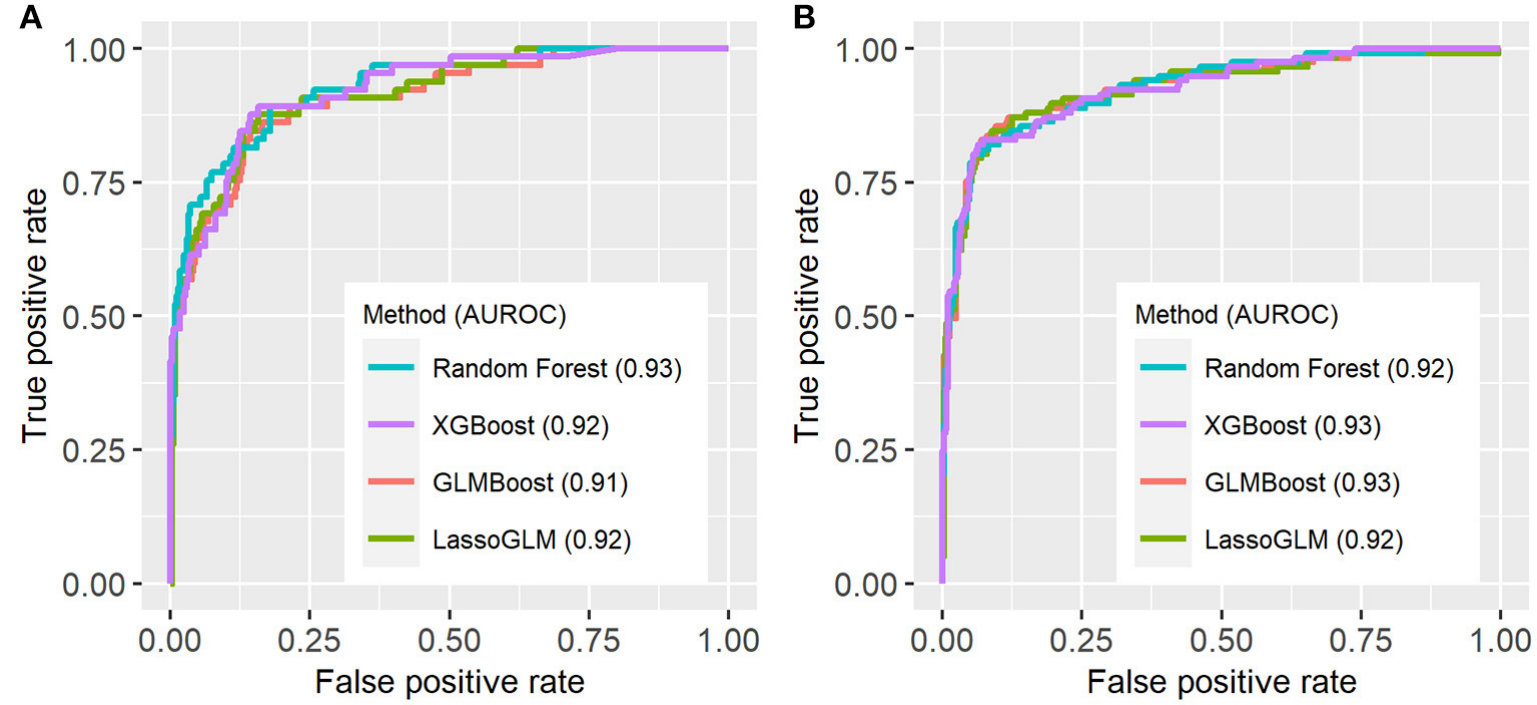
Receiver operating characteristic (ROC) curves for **(A)** IPSCC and **(B)** Proulx criteria for organ dysfunction. AUROC (area under ROC curve) values are indicated in the figure legend.

**Figure 5:**
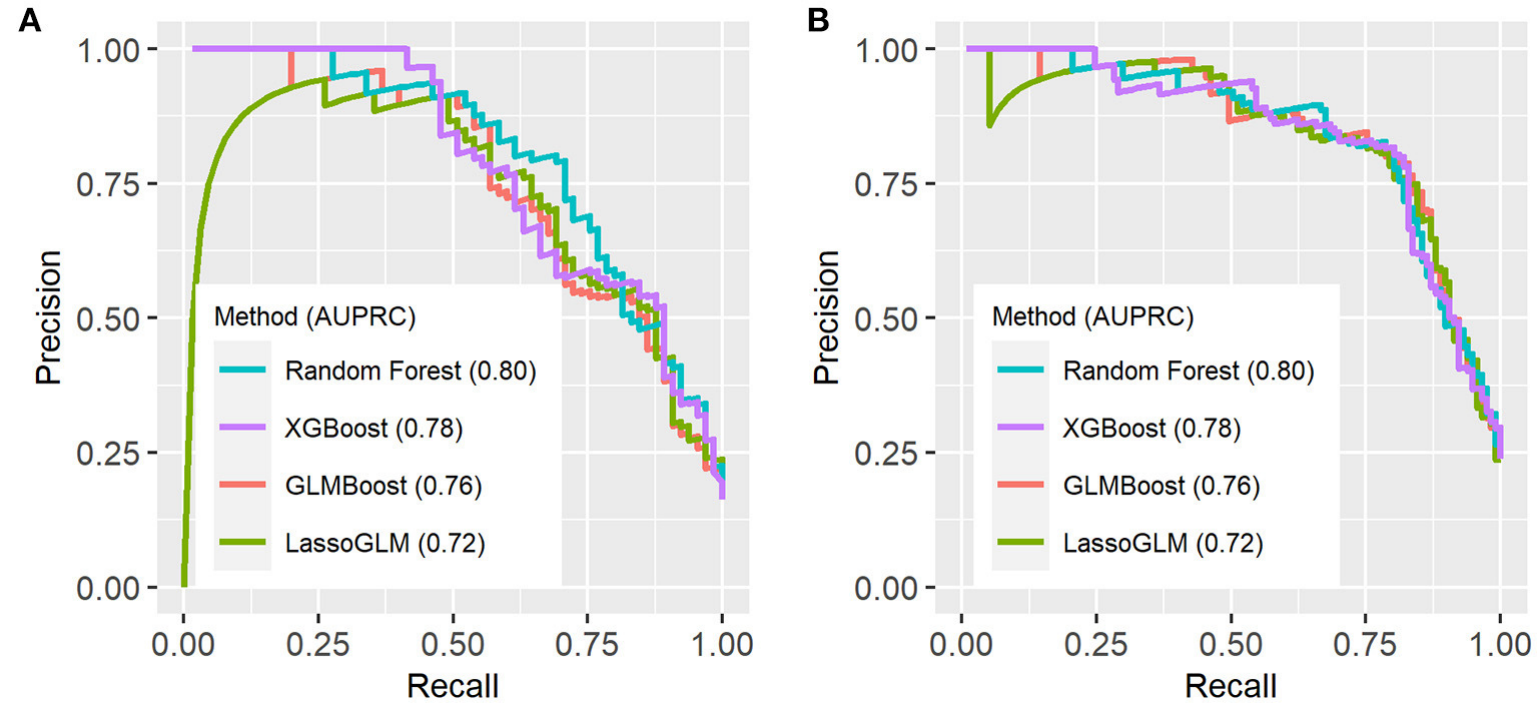
Precision-recall (PR) curves on test set using **(A)** IPSCC criteria and **(B)** Proulx et al. criteria for organ dysfunction. AUPRC (area under PR curve) values are indicated in the figure legend.

Figure 3 illustrates an example of HRA and associated EWT along with another example where the patient never developed MOD during the PICU stay (see Supplementary Figure 1 for more examples of time-varying risk scores). The mean [standard deviation (SD)] of AUC on the cross-validation folds were 0.89 (0.04), 0.89 (0.05), 0.89 (0.04), and 0.90 (0.04) (IPSCC criteria) and 0.93 (0.02), 0.93 (0.02), 0.92 (0.02), and 0.93 (0.02) (Proulx criteria) for XGBoost, random forest, GLMBoost, and LassoGLM methods, respectively. All cross-validation set performance metrics obtained using tuned model parameters are shown in Supplementary Figures 2, 3. The final model trained on the entire training set using tuned parameters that maximized cross-validation set performance and yielded AUC 0.92, 0.93, 0.91, and 0.92 (IPSCC criteria) and 0.92, 0.93, 0.91, and 0.92 (Proulx criteria) with XGBoost, random forest, GLMBoost, and LassoGLM methods, respectively, on the test set. All results discussed below refer to metrics evaluated on the test set unless specified otherwise. The ROC and precision-recall curves for all methods are shown in Figures 4, 5, respectively. The performance metrics for all methods are presented in Table 2. All four methods exhibited high specificity, with moderate sensitivity related to the low prevalence of MOD transition events in the data set. All models were able to learn a high degree of separation between the positive and negative classes reflected by the high AUC values (>0.91). Performance metrics were comparable across the four methods when maximum F1-score was used for optimal threshold detection. The highest positive predictive value (PPV) was obtained using the LassoGLM method (PPV, 0.72) for IPSCC criteria and XGBoost, GLMBoost, and LassoGLM methods (PPV, 0.81 for each method) for Proulx criteria, respectively, with maximum F1-score used for optimal threshold determination. Maximum F1-score criteria for threshold selection yielded consistently higher PPV than the point nearest (0, 1) on the ROC curve. The high negative predictive values (>0.92 for both IPSCC and Proulx criteria) for all methods indicate a low false alarm rate. Random forest (0.71) and XGBoost (0.81) were the best models in terms of maximum F1-score obtained using IPSCC and Proulx criteria, respectively. Precision-recall plot showed that the sensitivity could be boosted to ≥0.9 at the cost of reduced PPV ( ≤ 0.5) and *vice versa* (Figure 5).

**Table 2:** Performance metrics evaluated on test set for IPSCC and Proulx et al. criteria of multiple organ dysfunction.

**Metrics**	**Random forest**		**XGBoost**		**GLMBoost**		**LassoGLM**	
	**Nearest from (0, 1)^**a**^**	**Max F1-score^**a**^**	**Nearest from (0, 1)^**a**^**	**Max F1-score^**a**^**	**Nearest from (0, 1)^**a**^**	**Max F1-score^**a**^**	**Nearest from (0, 1)^**a**^**	**Max F1-score^**a**^**
**IPSCC criteria**								
Sensitivity	0.83	0.72	0.78	0.63	0.89	0.68	0.88	0.65
Specificity	0.84	0.94	0.88	0.94	0.76	0.93	0.82	0.95
Positive predictive value (PPV)	0.50	0.70	0.56	0.68	0.41	0.67	0.48	0.72
Negative predictive value (NPV)	0.96	0.95	0.95	0.93	0.97	0.94	0.97	0.93
Accuracy	0.84	0.90	0.86	0.89	0.78	0.89	0.83	0.90
Area under receiver operating characteristic curve (AUROC)	0.93		0.92		0.91		0.92	
**Proulx criteria**								
Sensitivity	0.85	0.82	0.82	0.80	0.85	0.79	0.85	0.77
Specificity	0.87	0.92	0.93	0.94	0.89	0.94	0.89	0.94
Positive predictive value (PPV)	0.68	0.76	0.78	0.81	0.70	0.81	0.70	0.81
Negative predictive value (NPV)	0.95	0.94	0.94	0.94	0.95	0.93	0.95	0.93
Accuracy	0.87	0.89	0.90	0.91	0.88	0.91	0.88	0.90
Area under receiver operating characteristic curve (AUROC)	0.93		0.92		0.93		0.92	

a *Nearest from (0,1) on ROC curve and maximum F1 score are two different criteria used for threshold selection.*

Supplementary Figure 4 shows the histograms of EWT across all four methods for the two sets of MOD criteria. The median EWTs for true positives detected by random forest, XGBoost, GLMBoost, and LassoGLM were 22.7, 29.8, 28.3, and 28.5 h for IPSCC criteria and 35.4, 37.0, 32.1, and 35.5 h for the Proulx criteria, respectively. The histograms of EWT normalized by the length of available data (Supplementary Figure 5) show that the majority of positive predicted cases were detected with HRA at the first instance of available data. The observed HRA event relative to the length of available data was significantly different between true positives and false positives [median (95% confidence interval) of pairwise differences was −0.29 (−0.53, −0.10), p <0.001 for random forest with IPSCC criteria, and −0.12 (−0.19, −0.06), p <0.001 for XGBoost with Proulx criteria using Wilcoxon rank sum test], and usually, HRA occurred relatively sooner in true positives for all methods as well as for both criteria. The HRIs for more than 64% of true positives were the same as their EWTs, implying that the risk scores stayed above the threshold after HRA for those cases. The distribution of HRI normalized by EWT was significantly different between true positives and false positives [median (95% confidence interval) of pairwise differences was 0 (−0.52, 0), p < 0.001 for random forest with IPSCC criteria, and −0.02 (−0.46, 0) for XGBoost with Proulx criteria using Wilcoxon rank sum test] across all methods and for both MOD criteria (Supplementary Table 3).

### Risk Group Stratification

**Figure 6:**
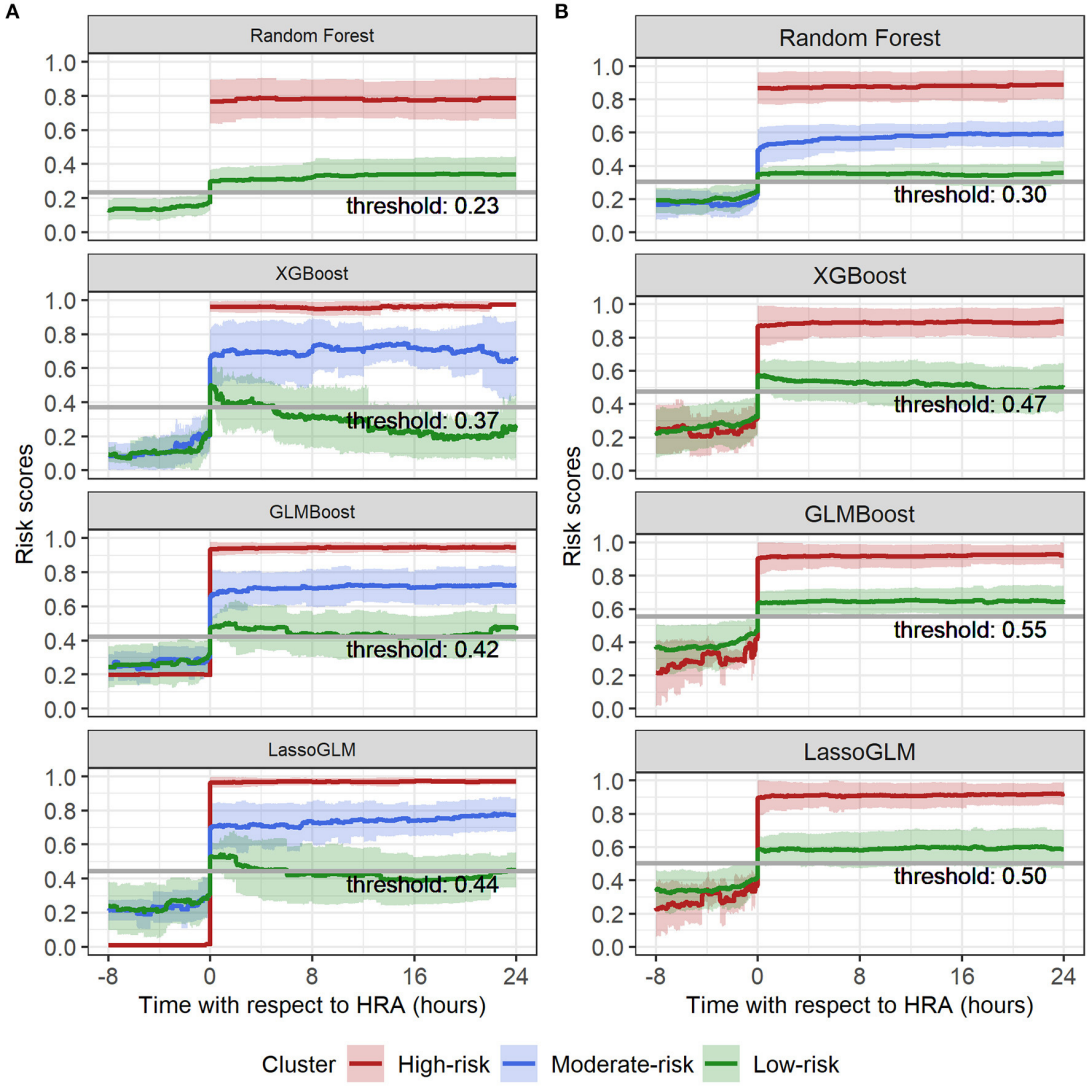
Spectral clustering on risk score trajectories for **(A)** IPSCC and **(B)** Proulx criteria. Solid lines indicate mean, and shaded regions indicate mean ± standard deviation of risk score trajectories within each cluster.

Within the group of positive predictions, the risk scores of the true positives were consistently much higher than the threshold following the HRA event, while those of the false positives tended to be only marginally above threshold, rendering these cases less distinct from the true negatives than the true positives. We used time-evolving risk score trajectories from 24-h time windows following HRA from all positive predicted cases in our test set to perform spectral clustering. The positive predicted risk score trajectories separated into two clusters (labeled as high risk and low risk) or three clusters (labeled as high risk, moderate risk, and low risk), depending on the method and on the set of MOD criteria used (Figure 6). All methods for both IPSCC and Proulx criteria yielded PPV ≥0.93 in the high-risk cluster, emphasizing higher confidence in predictions within that group (Table 3). Time to observed HRA relative to the length of available data was significantly smaller [median (95% confidence interval) of pairwise differences was 0 (−0.02, 0), p < 0.001 for random forest with IPSCC criteria, −0.16 (−0.34, −0.03) for XGBoost with Proulx criteria using Wilcoxon rank sum test] in the high- and moderate-risk groups than the low-risk group for all methods except LassoGLM with IPSCC criteria (Supplementary Table 4). These risk groups appeared to follow similar patterns prior to HRA and showed a significant difference in their risk score trajectories immediately following HRA. This observation raised the intuitive question as to whether the instantaneous value of risk score at HRA was directly related to the accuracy of our predictions. We divided all positive predicted labels into four groups based on quartiles of risk scores at HRA and observed a monotonic increase in PPV with increasing average risk score at HRA across these quartiles for all models using IPSCC criteria and for GLMBoost and LassoGLM models using Proulx criteria (Supplementary Table 5). The PPV in the highest quartile for all methods was ≥0.93.

**Table 3:** Positive predictive value and early warning times across risk groups obtained using spectral clustering.

**Method**	**Positive predictive value**			**Early warning time (h)** ^**a**^		
	**High-risk group**	**Moderate-risk group**	**Low-risk group**	**High-risk group**	**Moderate-risk group**	**Low-risk group**
**IPSCC criteria**						
Random forest	1.00	–	0.50	21.08 (8.04–82.98)	–	26.24 (4.30–55.96)
XGBoost	1.00	0.73	0.35	18.04 (7.55–75.95)	47.29 (19.44–177.47)	44.22 (10.05–57.02)
GLMBoost	1.00	0.74	0.37	38.58 (11.27–105.07)	35.00 (6.17–76.14)	25.93 (12.60–42.02)
LassoGLM	0.95	0.89	0.32	49.98 (13.38–124.17)	22.48 (4.80–67.40)	37.93 (24.49–48.27)
**Proulx criteria**						
Random forest	1.00	0.81	0.53	30.05 (10.06–75.47)	35.88 (19.98–62.46)	47.83 (17.25–145.86)
XGBoost	0.94	–	0.65	29.57 (14.31–68.12)	–	53.10 (17.66–127.60)
GLMBoost	0.93	–	0.71	30.67 (15.65–84.71)	–	36.63 (17.47–90.45)
LassoGLM	0.94	–	0.69	30.67 (16.58–97.55)	–	37.48 (17.45–90.70)

a *Early warning time presented as median (25th−75th percentile).*

*XGBoost, extreme gradient boosting; random forest, random forest-based probability machines; GLMBoost, gradient boosted generalized linear models; LassoGLM, L1-regularized generalized linear models*.

### Feature Importance

Our models were trained using a large number of features, and each machine learning method employed a different feature selection scheme. We found that a small subset of features had a relatively high contribution to these models. Supplementary Tables 6, 7 show a list of the 20 most important features with their relative importance metric scaled to range 0–100 in each model and for each set of MOD criteria.

## Discussion

### Predictive Power of Prognostic Models

The objective of this study was to explore suitable machine learning methods to build prognostic models for early identification of increased risk of transition from a no OD or single-organ dysfunction state to MOD in the general PICU population. The machine learning methods used in this study were selected based on the amount of available training data and the ability to tailor feature selection. All four methods achieved comparable and appreciable results for both diagnostic criteria for MOD. Maximum F1-score was preferred over the point nearest (0, 1) on the ROC curve for optimal threshold determination because it maximizes sensitivity (recall) and PPV (precision) instead of specificity in order to account for the class imbalance between target and control cases. AUC for all methods being higher than 0.9 for both IPSCC and Proulx criteria implied that our trained models were able to distinguish with high degree of accuracy data prior to MOD transition vs. data from no-MOD admission cases.

The considerably large median EWT indicates that our proposed models could alert physicians almost a day in advance for most MOD cases in PICU allowing sufficient time for early therapeutic intervention. The relatively low temporal variation in risk score trajectories is primarily due to low underlying physiologic data measurement frequencies, especially laboratory tests, which are normally performed in intervals of several hours and sometimes only once or twice a day. The sustained high-risk scores post-HRA serves as evidence that the probability of developing MOD can be used as a continuous indicator of severity over time. Models built for Proulx criteria demonstrates that a larger number of target samples can significantly boost the sensitivity without impacting the specificity and, hence, also yields slightly higher PPV.

### Risk Group-Based Prediction

Risk-group stratification among positive predicted labels was pivotal in assigning confidence to the model predictions. We chose spectral clustering over more conventional clustering algorithms such as k-means to avoid assuming homogeneity in the density of different clusters. The risk stratification method was highly effective in identifying the high-risk group (≥93% prediction accuracy) and, therefore, could be used as an additional alert for severe cases with extremely high likelihood of developing MOD. Moreover, the risk score trajectories of high-risk groups showed very high (0.7–1.0), non-decreasing, and steady evolution of risk scores over time and exhibited tighter clustering around mean tendency than the moderate- and low-risk groups.

Due to the observed large separation in the risk score trajectories at HRA across different risk groups, we were able to also separate the patients into groups based on quartiles of their risk scores at HRA. The PPV in these four groups were monotonically increasing with increasing average risk in each group, and the highest quartile yielded 100% PPV for all methods (except Lasso-GLM with IPSCC criteria) and both sets of diagnostic criteria. Therefore, in addition to HRA predictions, assigning confidence based on the risk group assignment would reduce the burden of false alarms and allow patient severity monitoring.

### Organ Dysfunction Labels

The observed rate of MOD during the PICU stay obtained using IPSCC and Proulx criteria (36.2 vs. 28.1%) was similar to the results presented by Villeneuve et al. (1) (37.3 vs. 21.4%, respectively). The discordance in MOD rates is primarily attributed to different rates of cardiovascular and neurologic dysfunction. Notably, the continuous binary MOD labels generated using IPSCC criteria had higher temporal stability than the Proulx criteria.

### Feature Selection and Importance

Conventional regression-based methods suffer from multiple correlated variables and require manual feature pruning or regularized regression. We took advantage of the built-in feature selection process of the methods used for training. Decision tree-based methods like random forest and XGBoost are ensembles of multiple decision trees that use a random subset of relevant features and a specified maximum depth to prune features. GLMBoost uses gradient boosting with component-wise linear models, which prunes features by restricting the number of boosting iterations and selecting only one feature in each iteration. LassoGLM is an L1-regularized version of a generalized linear model, which suppresses the smaller weight features in the model and retains only the most significant ones. Some procedures (e.g., arterial catheter placement) or laboratory tests are performed by clinicians only for sicker patients, which makes the presence vs. absence of a variable an important predictor as well. We added a missingness indicator for all laboratory test results and for placement of an arterial catheter. This concept was previously endorsed in work by Sharafoddini et al. (37).

Patient vital signs (e.g., core vs. peripheral temperature difference) as well as specific laboratory test results [e.g., prothrombin time (PT), activated partial thromboplastin time (aPTT), arterial pH, pCO_2_, and red blood cell distribution width] and interventions (e.g., red blood cell transfusion volume, inotrope use) featured prominently on the list of the 20 most important features for each machine learning method and for both sets of MOD criteria. One caveat is that these methods are prone to inconsistency in feature importance among correlated features, and unlike in linear models, the feature importance of non-linear decision tree ensemble methods are not additive. We observed higher concordance in feature rankings among non-linear models (random forest and XGBoost) and among linear models (GLMBoost and Lasso-GLM), but lower agreement between linear vs. non-linear models. This difference in feature ranking can be attributed to the difference in learning paradigms of these models; random forest and XGBoost combines predictions from an ensemble of decision trees, whereas GLMBoost and Lasso-GLM are regression-based linear methods. The small differences in the relative ranking of features among the decision tree-based methods can be explained by the random sampling of features in each iteration. Many of the 20 most important features overlap with those used in the MOD diagnostic criteria, but interestingly, we also observed several outside of MOD diagnostic criteria, including red blood cell distribution width, core vs. peripheral temperature difference, patient weight and age, arterial bicarb, alkaline phosphatase, C-reactive protein, monocyte number and percent, eosinophil number and percent, mean corpuscular volume, serum calcium and ionized calcium, aspartate aminotransferase, urine pH, urine color, glucose, body temperature, and AHG antibody screening. Future larger studies should be undertaken to understand the contribution of each of these features to the pathophysiology and evolution of MOD.

## Limitations

This study was limited to data from a single mixed PICU, and multicenter studies would be needed to evaluate generalizability. A larger dataset would also allow for comparison of prediction performance across different organ system failure transitions such as respiratory to respiratory plus neurologic, etc. Another limitation of this study includes latency in reporting of non-point-of-care laboratory test results, which may alter the exact time of observed organ dysfunction. Additionally, our models were unable to use PICU day 1 MOD transitions due to the absence of pre-PICU admission laboratory results and 24-h retrospective nature of the organ dysfunction definitions. We also recognize our limitation of not being able to compare the model's early prediction ability with a clinician's ability to identify the likelihood of transition to MOD state due to the retrospective nature of our observational study. Therefore, comparing the predictive ability of prediction models with live clinical decision making should be explored in future validation studies.

### Considerations for the Use of Early Prediction Models in Clinical Settings

Artificial intelligence (AI)-based healthcare applications constitute an evolving and rapidly expanding field aiming to address a large number of important clinical questions by identifying patterns in data that are either too subtle for clinicians to see or are “hiding in plain sight” and often missed (38). AI applications have focused on early diagnosis, prediction of clinical outcomes, and treatment recommendations (39–46). AI-based models can condense physiologic and laboratory monitoring data to informative and explainable composite quantitative scores to help clinicians monitor the patient's severity of illness as well as provide timely insights into future clinical outcomes. Obviously, clinicians cannot treat what they do not recognize; we posit early recognition will allow treatment when organ dysfunction is less severe and potentially more easily reversible. The spectral clustering-based risk stratification method could further allow clinicians to identify a high-risk group with high accuracy, fostering efficient, and timely patient management. Future studies are needed to study the impact of therapeutic interventions on the patient's risk for developing individual as well as MOD. The roadmap to deploying these models in real-time bedside monitoring algorithms should include rigorous validation (47) keeping in consideration the legal, social, and economic implications of AI in healthcare (45, 48).

## Conclusions

Our prognostic model-building approach has led to the development of a set of models with demonstrated ability to predict high-risk alerts in patients who transitioned into a MOD state. The risk stratification methodologies have shown ≥93% positive predictive value in the highest risk groups. Therefore, this combined approach of continuous time risk monitoring, early warning of development of MOD, and risk group stratification could significantly aid in the monitoring and ultimately the management of critically ill children.

## Data Availability Statement

The datasets presented in this article are not readily available because the dataset contains identifiable patient health information. Requests to access the datasets should be directed to mbembea1@jhmi.edu.

## Ethics Statement

The studies involving human participants were reviewed and approved by The Johns Hopkins Institutional Review Board. Written informed consent from the participants' legal guardian/next of kin was not required to participate in this study in accordance with the national legislation and the institutional requirements.

## Author Contributions

SB performed data cleaning, model building, statistical analysis, interpretation of prediction results and prepared first draft of the manuscript. JG and SS contributed to result interpretation, providing feedback on model building, result interpretation and manuscript revision. JF provided insights on clinical relevance and interpretation of prediction outcomes. MB and RW served as principal investigators of the study and contributed to the study design and direction, interpretation of results, and final drafting of the manuscript. All authors reviewed the final draft of the manuscript and approved for submission.

## Conflict of Interest

The authors declare that the research was conducted in the absence of any commercial or financial relationships that could be construed as a potential conflict of interest.

## Publisher's Note

All claims expressed in this article are solely those of the authors and do not necessarily represent those of their affiliated organizations, or those of the publisher, the editors and the reviewers. Any product that may be evaluated in this article, or claim that may be made by its manufacturer, is not guaranteed or endorsed by the publisher.
