# Supplementary material for: Early Prediction of Multiple Organ Dysfunction in the Pediatric Intensive Care Unit
Source: Front Pediatr. 2021 Aug 16;9:711104. doi: 10.3389/fped.2021.711104 (PMC8415553; doi:10.3389/fped.2021.711104)
Supplement: Supplementary file 1 [file Data_Sheet_1.docx]

**Early Prediction of Multiple Organ Dysfunction in the Pediatric Intensive Care Unit**

Sanjukta N. Bose, Joseph L. Greenstein, James C. Fackler, Sridevi V. Sarma, Raimond L. Winslow, Melania M. Bembea

Supplementary Material

**Table of Contents**

| **Content** | **Pages** |
| --- | --- |
| **Supplementary Methods** | 2-3 |
| **Supplementary Figure 1.** Examples of time evolving risk-score trajectories for positive predictions (A-D) and negative predictions (E-H). | 4 |
| **Supplementary Figure 2.** Cross-validation performance on training set using IPSCC organ dysfunction criteria. | 5 |
| **Supplementary Figure 3.** Cross-validation performance on training set using Proulx et al. organ dysfunction criteria. | 6 |
| **Supplementary Figure 4.** Histogram of early warning times in hours for (A) IPSCC and B. Proulx et al. criteria. | 7 |
| **Supplementary Figure 5.** Histogram of early warning times normalized by length of available data in true positive cases for (A) IPSCC and (B) Proulx et al. criteria. | 8 |
| **Supplementary Table 1.** List of all features used for building models | 9-14 |
| **Supplementary Table 2.** Patient characteristics by multiple organ dysfunction status based on Goldstein et al. and Proulx et al. criteria | 15-18 |
| **Supplementary Table 3.** Distribution of high risk intervals (HRI) normalized by length of available data in positive predictions for all methods using both Goldstein and Proulx criteria | 19 |
| **Supplementary Table 4.** Early warning times (EWT) relative to length of available data across high and moderate risk group vs. low risk group based on spectral clustering of risk-score trajectories of positive predictions for all methods using both Goldstein and Proulx criteria | 20 |
| **Supplementary Table 5.** Positive predictive value across quartiles of risk-scores for positive predicted cases immediately following high-risk alert events | 21 |
| **Supplementary Table 6.** Table of 20 most important features obtained from each of the four methods using Goldstein et al. organ dysfunction criteria | 22 |
| **Supplementary Table 7.** Table of 20 most important features obtained from each of the four methods using Proulx et al. organ dysfunction criteria. | 23 |
| **References** | 24 |

# Supplementary Methods

## **Features**

A total of 7 vital sign variables including heart rate, respiratory rate, SpO2, systolic and diastolic non-invasive blood pressure and systolic and diastolic invasive blood pressure measurements from arterial catheter were available as direct downloads from the bedside monitors. Heart rate, respiratory rate, SpO2 and invasive blood pressure measurements were available at 1-minute intervals whereas non-invasive blood pressure measurements were available infrequently at an average of 15 minute intervals. Invasive blood pressure measurements were present in only 27% of included PICU admissions as opposed to other vital signs which were present in more than 99% of included PICU admissions. We added a missingness indicator variable to indicate 1 if arterial catheter was absent or 0 if present in a patient, to reflect clinicians’ decision that an arterial catheter was warranted for hemodynamic monitoring or for frequent blood sampling. Infrequently stored vital signs data from electronic medical records (EMR) were matched with the selected minute-to-minute vital sign data in order to verify the degree of concordance between recorded data from two sources. Additional model features were extracted from EMR flowsheets, including demographics (age, sex, race, weight, procedures), other vital signs (body temperature [core – peripheral], interventions such as medication infusions (vasoactive drugs, sedatives, neuromuscular blockers), respiratory support, transfusion type and volume per kg weight, renal replacement therapy (hemofiltration, peritoneal dialysis) and laboratory test results.

In order to facilitate building a well generalizable model for our dataset, all laboratory test results which were measured in at least 25% of the included PICU admissions were used as input features. Since some of the laboratory tests are only ordered for patients with existing conditions and comorbidities, missingness indicator corresponding to each laboratory result variable was added to the feature set as it bears potentially relevant information about patient severity status. The prognostic value in adding missingness indicator for laboratory result data has also been emphasized in a recent work by Sharafoddini et al.(1) Carry forward interpolation was used for all features which were measured less frequently than every minute and median interpolation was applied to features measured every minute. Individual features were rescaled to range (0, 1) to facilitate interpretation of coefficients in regression models. A complete list of all features used in the models is presented in Supplementary Table 1.

## Performance metrics

Let, TP, FP, TN, FN denote the number of true positives, false positives, true negatives, and false negatives, respectively. The definition for each of the performance metrics used in this study are listed below.

**AUROC:** Area under the receiver operating characteristic (ROC) curve, where ROC is obtained by plotting true positive rate vs. false positive rate for different values of threshold used by a binary classifier. This metric is also known as C-statistic and is commonly used as a metric to quantify a binary classifier’s diagnostic ability. AUROC value ranges between 0-1. Higher values of AUROC indicate a better classifier where the AUROC of a random classifier is 0.5.

**Sensitivity:** It is also called true positive rate or, recall. It is computed as $\frac{TP}{TP+FN}$, Higher sensitivity indicates a higher proportion of positive samples are classified correctly.

**Specificity:** It is same as (1 – false positive rate) and is computed as $\frac{TN}{TN+FP}$. Higher specificity indicates a higher proportion of negative samples are classified correctly.

**Accuracy:** It is computed as $\frac{TP+TN}{N}$, where N is the total number of samples $\left( N= TP+FP+TN+FN \right).$

**Positive Predictive Value (PPV):** It is also known as precision. It is computed as $\frac{TP}{TP+FP}$ . Higher values PPV indicate a higher proportion of all positive predictions are correct.

**Negative Predictive Value (NPV):** It is computed as $\frac{TN}{TN+FN}$ . Higher values NPV indicate a higher proportion of all negative predictions are correct.

**F1-score:** It is also known as balanced F-score. It is computed as $\frac{2}{sensitivity^{-1}+PPV^{-1}}= \frac{2*TP}{2*TP+FP+FN}$. Maximizing F1-score strikes a balance between maximizing sensitivity and PPV by weighting them equally.

# Supplementary Figures and Tables

## Supplementary Figures


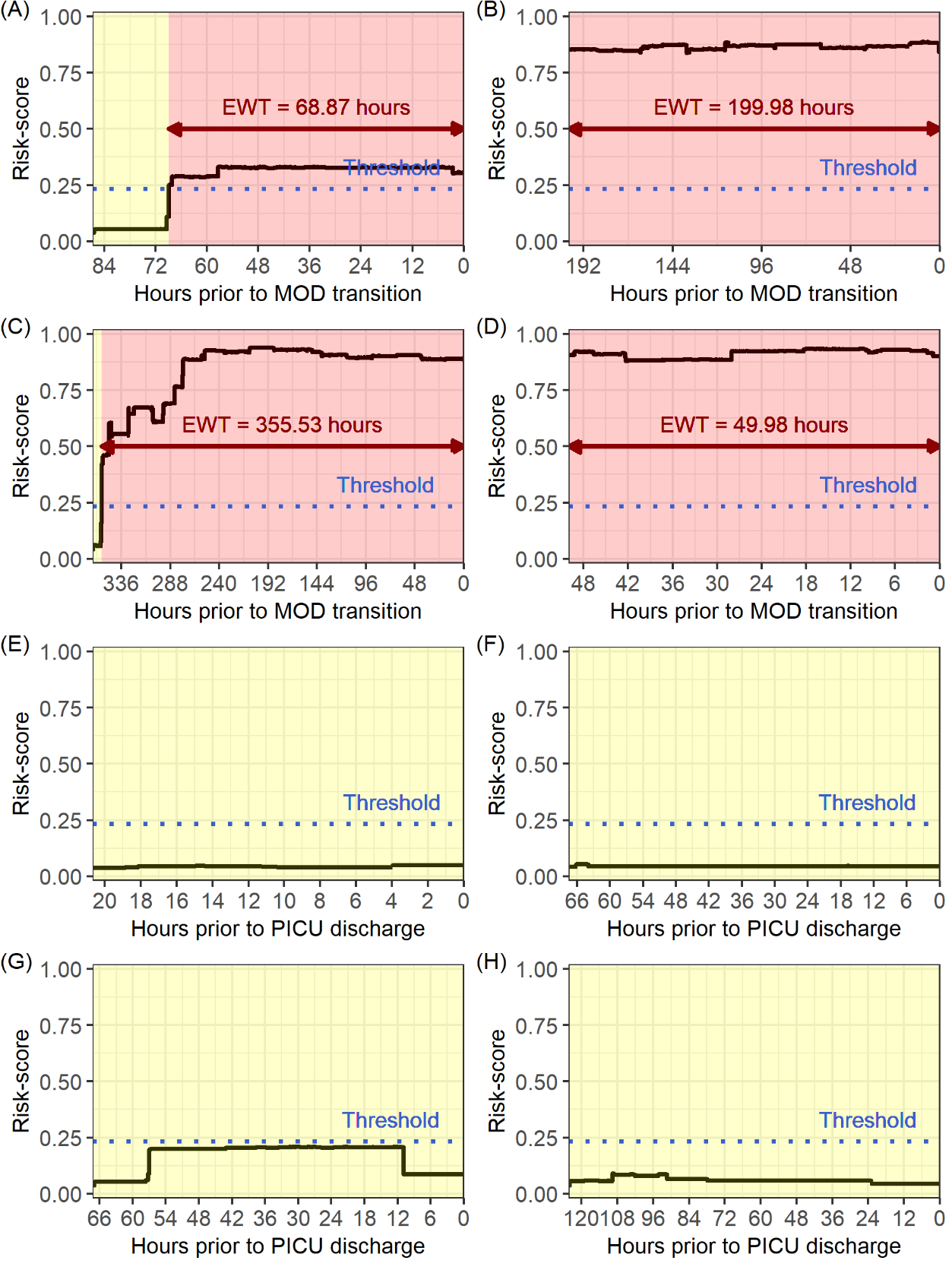


**Supplementary Figure 1.** Examples of time evolving risk-score trajectories for positive predictions (A-D) and negative predictions (E-H).


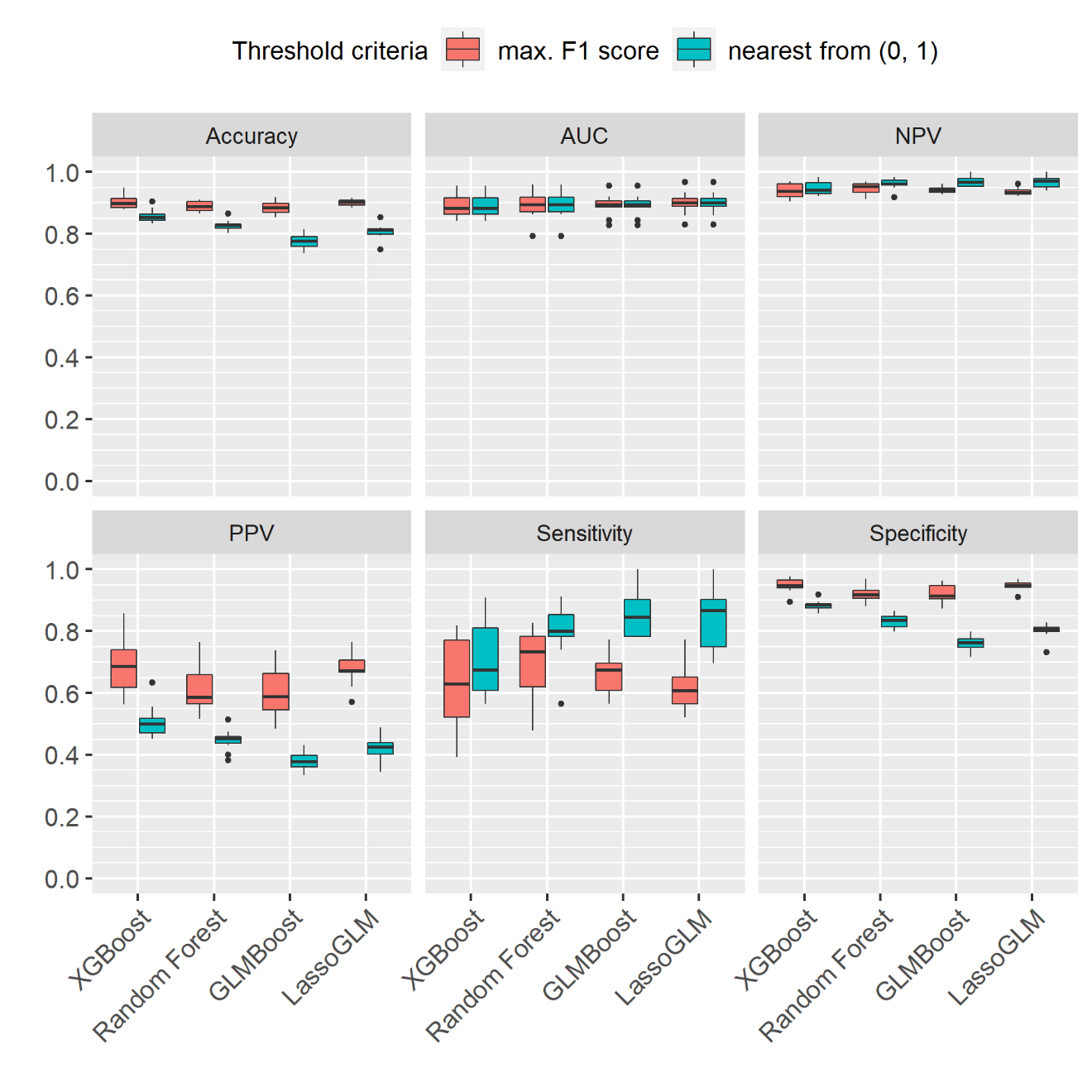
**Supplementary Figure 2.** Cross-validation performance on training set using IPSCC organ dysfunction criteria.


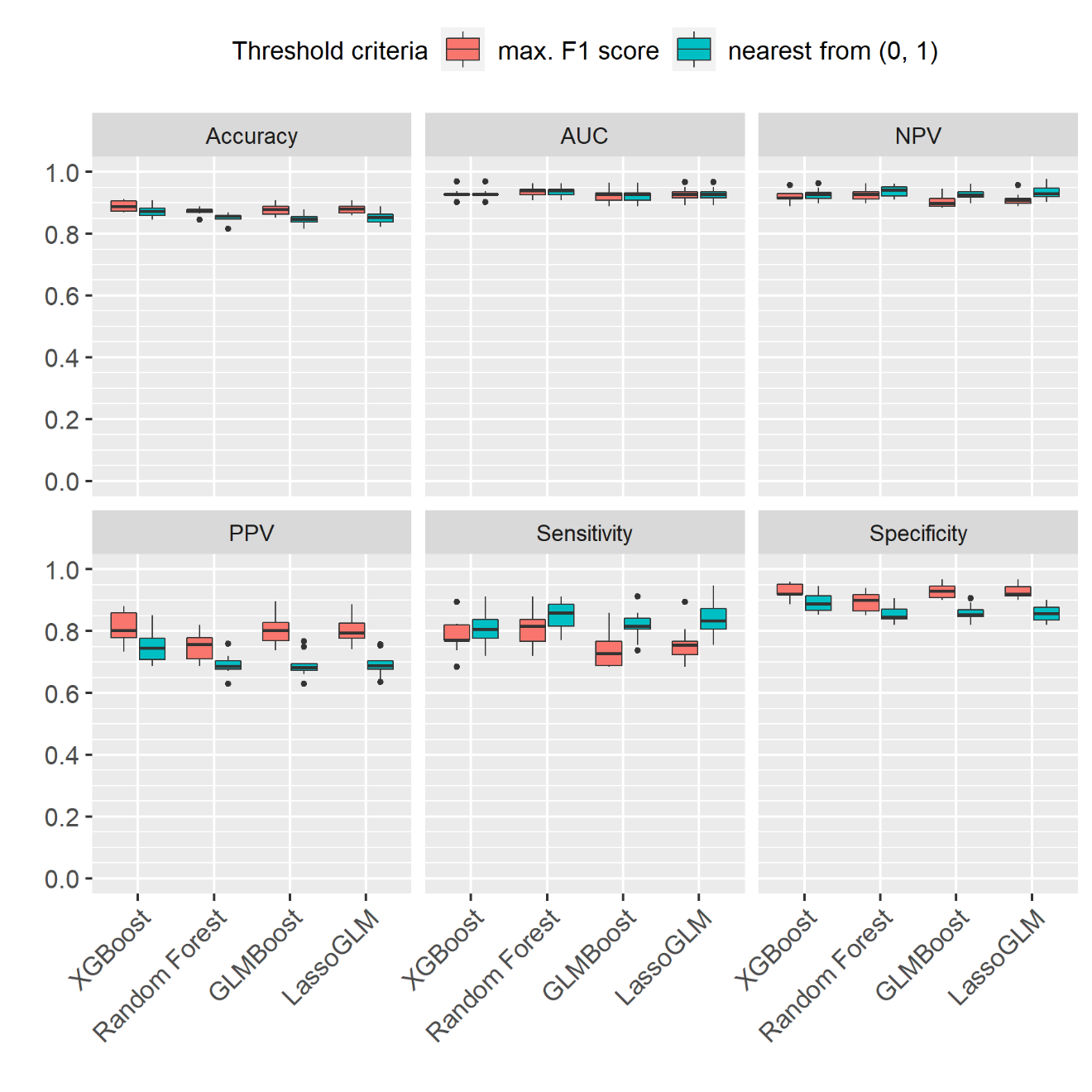


**Supplementary Figure 3.** Cross-validation performance on training set using Proulx et al. organ dysfunction criteria.


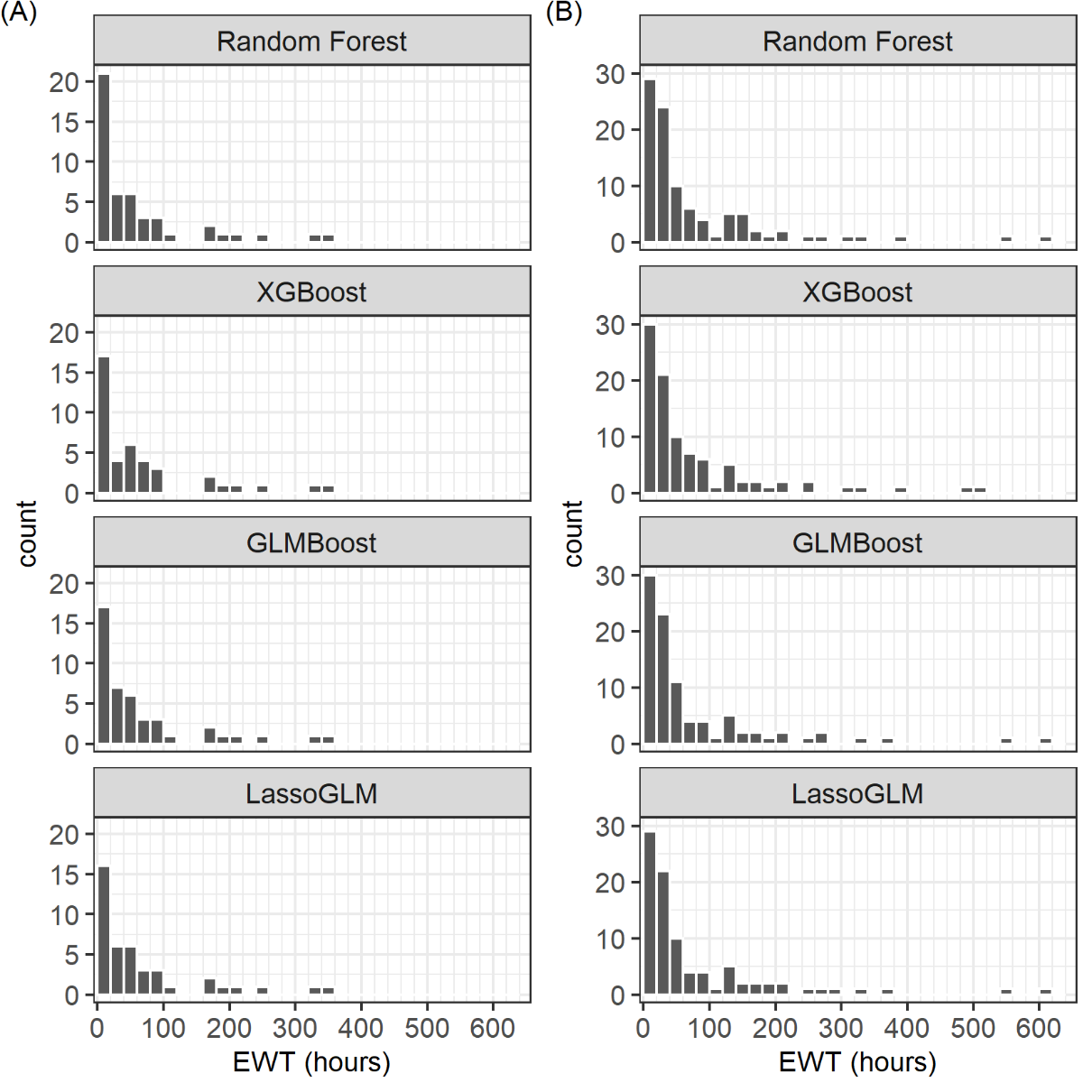


**Supplementary Figure 4.** Histogram of early warning times in hours for (A) IPSCC and B. Proulx et al. criteria.


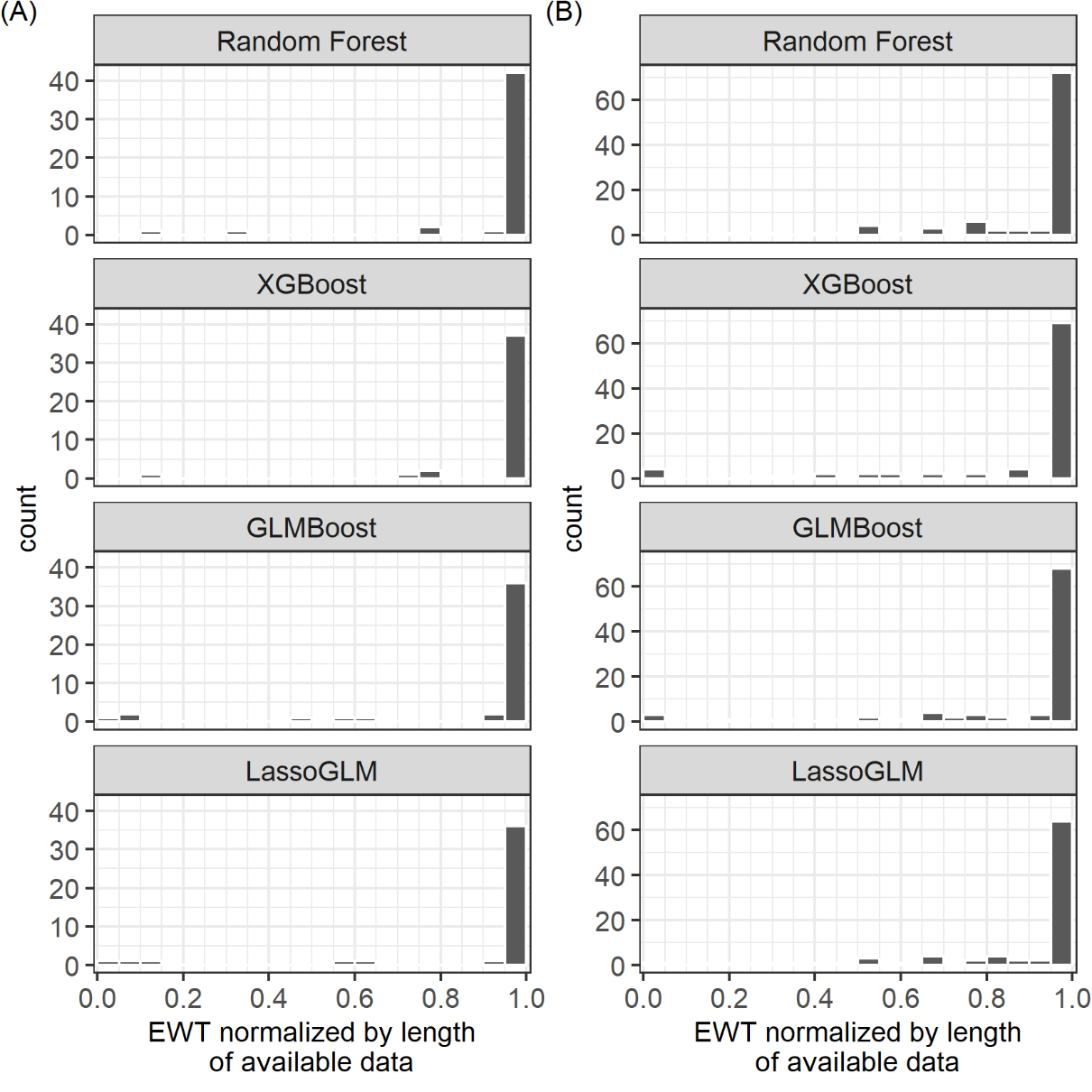


**Supplementary Figure 5.** Histogram of early warning times normalized by length of available data in true positive cases for (A) IPSCC and (B) Proulx et al. criteria.

## Supplementary Tables

**Supplementary Table 1.** List of all features used for building models

| Feature | Time varying | Type | Units | Description | Source |
| --- | --- | --- | --- | --- | --- |
| Age | False | Numeric | years | Age at PICU admission calculated using date of birth | EMR – demographics |
| Gender | False | Categorical | - | 1 = Male, 0 = female | EMR – demographics |
| Race | False | Categorical | - | Unique values: White/African American/Other | EMR - demographics |
| HR | True | Numeric | Beats per minute | Heart rate | Bedside monitor data |
| HRSD5 | True | Numeric | Beats per minute | Standard deviation in heart rate measured in sliding 5-minute windows | Bedside monitor data |
| HRSD60 | True | Numeric | Beats per minute | Standard deviation in heart rate measured in sliding 60-minute windows | Bedside monitor data |
| RR | True | Numeric | Breaths per minute | Respiratory rate | Bedside monitor data |
| NBP_S | True | Numeric | mmHg | Systolic blood pressure (non-invasive) | Bedside monitor data |
| NBP_D | True | Numeric | mmHg | Diastolic blood pressure  (non-invasive) | Bedside monitor data |
| ABP_S*^a^* | True | Numeric | mmHg | Systolic blood pressure (arterial line) | Bedside monitor data |
| ABP_D*^a^* | True | Numeric | mmHg | Diastolic blood pressure  (arterial line) | Bedside monitor data |
| SpO2_pcent | True | Numeric | % | Pulse oximetry | Bedside monitor |
| Weight | False | Numeric | kg | Weight measured at PICU admission | EMR – vital signs |
| Temperature | True | Numeric | °C | Body temperature(2) = core temperature or peripheral temperature + 1°C | EMR – vital signs |
| Temp_diff | True | Numeric | °C | Core to peripheral temperature difference | EMR – vital signs |
| GCS | True | Numeric | - | Glasgow Coma Score | EMR – assessments flowsheet |
| IS | True | Numeric | - | Inotrope score = dopamine dose (μg/kg/min) + dobutamine dose (μg/kg/min) + 100 x epinephrine dose (μg/kg/min) | EMR – vital signs |
| VIS | True | Numeric | - | Vasoactive-inotrope score(3) = IS + 10 x milrinone dose (μg/kg/min) + 10000 x vasopressin dose (U/kg/min) + 100 x norepinephrine dose (μg/kg/min) | EMR – vital signs |
| PaO2 | True | Numeric | mmHg | Arterial partial pressure of oxygen | EMR – respiratory + lab results flowsheets |
| PaCO2 | True | Numeric | mmol/L | Arterial partial pressure of carbon dioxide | EMR – respiratory + lab results flowsheets |
| OI | True | Numeric | - | Oxygenation Index(4–6) = (FiO2 x mean airway pressure x 100)/PaO2. OI is only measured when a patient is on mechanical ventilation. | EMR – respiratory + lab results flowsheets |
| OSI | True | Numeric | - | Oxygen saturation index(4–6) = (FiO2 x mean airway pressure x 100)/SpO2. OSI is only measured when a patient is on mechanical ventilation. | EMR – respiratory + lab results flowsheets |
| P2F | True | Numeric | - | PaO­_2_ to FiO_2_ ratio (only measured when patient is on mechanical ventilation or non-invasive positive pressure ventilation) | EMR – respiratory + lab results flowsheets |
| S2F | True | Numeric | - | SpO_2_ to FiO_2_ ratio (only measured when patient is on mechanical ventilation or non-invasive positive pressure ventilation) | EMR – respiratory + lab results flowsheets |
| Resp_supp | True | Categorical | - | Respiratory support. Unique values = MV (mechanical ventilation), NIPPV (Non-invasive positive pressure ventilation), Suppl_O2 (supplemental oxygen without pressure), RA (room air) | EMR - respiratory flowsheet |
| ECMO | True | Numeric (binary) | - | Extracorporeal Membrane Oxygenation. 1 = received, 0 = did not receive | EMR – respiratory flowsheet |
| RBC_transfusion_rate*^b^* | True | Numeric | mL/kg/min | Red blood cell transfusion rate*^b^* | EMR – input-output flowsheet |
| FFP_transfusion_rate*^b^* | True | Numeric | mL/kg/min | Fresh frozen plasma transfusion rate*^b^* | EMR – input-output flowsheet |
| Platelet_transfusion_rate*^b^* | True | Numeric | mL/kg/min | Platelet transfusion rate*^b^* | EMR – input-output flowsheet |
| Cryoppt_transfusion_rate*^b^* | True | Numeric | mL/kg/min | Cryoprecipitate transfusion rate*^b^* | EMR – input-output flowsheet |
| RBC_transfusion_vol*^c^* | True | Numeric | mL | Red blood cell transfusion volume since PICU admission*^c^* | EMR – input-output flowsheet |
| FFP_transfusion_vol*^c^* | True | Numeric | mL | Fresh frozen plasma transfusion volume since PICU admission*^c^* | EMR – input-output flowsheet |
| Platelet_transfusion_vol*^c^* | True | Numeric | mL | Platelet transfusion volume since PICU admission*^c^* | EMR – input-output flowsheet |
| Cryoppt_transfusion_vol*^c^* | True | Numeric | mL | Cryoprecipitate transfusion volume since PICU admission*^c^* | EMR – input-output flowsheet |
| Hem_dialysis | True | Numeric (binary) | - | Hemofiltration. 1 = received, 0 = did not receive. | EMR – input-output flowsheet |
| Peri_dialysis | True | Numeric (binary) | - | Peritoneal dialysis. 1 = Received, 0 = did not receive. | EMR – input-output flowsheet |
| Lactate*^a^* | True | Numeric | mmol/L | Lactate | EMR – lab results flowsheet |
| Platelet*^a^* | True | Numeric | K/mm^3^ | Platelet count | EMR – lab results flowsheet |
| PT*^a^* | True | Numeric | seconds | Prothrombin time | EMR – lab results flowsheet |
| aPTT*^a^* | True | Numeric | seconds | Activated prothrombin time | EMR – lab results flowsheet |
| Bilirubin*^a^* | True | Numeric | mg/dL | Bilirubin | EMR – lab results flowsheet |
| pH_art*^a^* | True | Numeric | - | Arterial pH | EMR – lab results flowsheet |
| pH_nonart*^a^* | True | Numeric | - | Non-arterial pH | EMR – lab results flowsheet |
| Na*^a^* | True | Numeric | mmol/L | Sodium | EMR – lab results flowsheet |
| K*^a^* | True | Numeric | mmol/L | Potassium | EMR – lab results flowsheet |
| Glucose*^a^* | True | Numeric | mg/dL | Blood glucose | EMR – lab results flowsheet |
| Creatinine*^a^* | True | Numeric | mg/dL | Creatinine (serum) | EMR – lab results flowsheet |
| BUN*^a^* | True | Numeric | mg/dL | Blood urea nitrogen | EMR – lab results flowsheet |
| ASTALTRatio*^a^* | True | Numeric | - | Aspartate aminotransferase to alanine aminotransferase ratio | EMR – lab results flowsheet |
| AbsoluteLymphocyteCount*^a^* | True | Numeric | K/mm^3^ | Absolute lymphocyte count | EMR – lab results flowsheet |
| AbsoluteNeutrophilCount*^a^* | True | Numeric | K/mm^3^ | Absolute neutrophil count | EMR – lab results flowsheet |
| AlanineAminoTransferase*^a^* | True | Numeric | units/L | Alanine aminotransferase | EMR – lab results flowsheet |
| AlkalinePhosphatase*^a^* | True | Numeric | units/L | Alkaline phosphatase | EMR – lab results flowsheet |
| AnionGap*^a^* | True | Numeric | mmol/L | Anion gap | EMR – lab results flowsheet |
| AspartateAminoTransferase*^a^* | True | Numeric | units/L | Aspartate aminotransferase | EMR – lab results flowsheet |
| BasophilPercent*^a^* | True | Numeric | % | Basophil percent | EMR – lab results flowsheet |
| Basophils*^a^* | True | Numeric | K/mm^3^ | Basophils | EMR – lab results flowsheet |
| Calcium*^a^* | True | Numeric | mg/dL | Calcium | EMR – lab results flowsheet |
| CO2*^a^* | True | Numeric | mmol/L | Carbon dioxide in blood | EMR – lab results flowsheet |
| CalculatedBicarbArterial*^a^* | True | Numeric | mmol/L | Calculated arterial bicarbonate | EMR – lab results flowsheet |
| CalculatedBicarbnonarterial*^a^* | True | Numeric | mmol/L | Calculated non-arterial bicarbonate | EMR – lab results flowsheet |
| Chloride*^a^* | True | Numeric | mmol/L | Chloride | EMR – lab results flowsheet |
| EosinophilPercent*^a^* | True | Numeric | % | Eosinophil percent | EMR – lab results flowsheet |
| EosinophilNumber*^a^* | True | Numeric | K/mm^3^ | Eosinophil Number | EMR – lab results flowsheet |
| Eosinophils*^a^* | True | Numeric | K/mm^3^ | Eosinophils | EMR – lab results flowsheet |
| EpithelialCells*^a^* | True | Numeric | #/HPF*^d^* | Epithelial Cells | EMR – lab results flowsheet |
| GranularCasts*^a^* | True | Numeric | #/HPF*^d^* | Granular Casts | EMR – lab results flowsheet |
| Hematocrit*^a^* | True | Numeric | % | Hematocrit | EMR – lab results flowsheet |
| HyalineCasts*^a^* | True | Numeric | #/HPF*^d^* | Hyaline Casts | EMR – lab results flowsheet |
| ImmatureGranPercent*^a^* | True | Numeric | % | Immature granular percent | EMR – lab results flowsheet |
| ImmatureGranNumber*^a^* | True | Numeric | K/mm^3^ | Immature granular number | EMR – lab results flowsheet |
| IonizedCalcium*^a^* | True | Numeric | mmol/L | Ionized calcium | EMR – lab results flowsheet |
| LymphNumber*^a^* | True | Numeric | K/mm^3^ | Lymphocyte number | EMR – lab results flowsheet |
| LymphPercent*^a^* | True | Numeric | % | Lymphocyte percent | EMR – lab results flowsheet |
| Lymphocytes*^a^* | True | Numeric | K/mm^3^ | Lymphocytes | EMR – lab results flowsheet |
| MagnesiumSerum*^a^* | True | Numeric | mg/dL | Serum magnesium | EMR – lab results flowsheet |
| MeanCorpusHGBConc*^a^* | True | Numeric | g/dL | Mean corpuscular HGB concentration | EMR – lab results flowsheet |
| MeanCorpuscularHGB*^a^* | True | Numeric | pg | Mean corpuscular HGB | EMR – lab results flowsheet |
| MeanCorpuscularVolume*^a^* | True | Numeric | fL | Mean corpuscular volume | EMR – lab results flowsheet |
| MeanPlateletVolume*^a^* | True | Numeric | fL | Mean platelet volume | EMR – lab results flowsheet |
| MonocyteNumber*^a^* | True | Numeric | K/mm^3^ | Monocyte number | EMR – lab results flowsheet |
| MonocytePercent*^a^* | True | Numeric | % | Monocyte percent | EMR – lab results flowsheet |
| Monocytes*^a^* | True | Numeric | K/mm^3^ | Monocytes | EMR – lab results flowsheet |
| NRBCNumber*^a^* | True | Numeric | K/mm^3^ | Nucleated red blood cell number | EMR – lab results flowsheet |
| NeutrophilNumberANC*^a^* | True | Numeric | K/mm^3^ | Neutrophil number | EMR – lab results flowsheet |
| NeutrophilPercent*^a^* | True | Numeric | % | Neutrophil percent | EMR – lab results flowsheet |
| Polymorphonuclear*^a^* | True | Numeric | K/mm^3^ | Polymorphonuclear leukocytes | EMR – lab results flowsheet |
| RBCCountUrine*^a^* | True | Numeric | #/HPF*^d^* | Red blood cell count in urine | EMR – lab results flowsheet |
| RBCDistributionWidth*^a^* | True | Numeric | % | Red blood cell distribution width | EMR – lab results flowsheet |
| RedBloodCellCount*^a^* | True | Numeric | M/mm^3^ | Red blood cell count | EMR – lab results flowsheet |
| RedBloodCells*^a^* | True | Numeric | #/HPF*^d^* | Red blood cells, urine | EMR – lab results flowsheet |
| SpecificGravity*^a^* | True | Numeric | - | Specific gravity, urine | EMR – lab results flowsheet |
| SpecimenpH*^a^* | True | Numeric | - | Specimen pH, urine | EMR – lab results flowsheet |
| WBCCountUrine*^a^* | True | Numeric | #/HPF*^d^* | White blood cell count, urine. | EMR – lab results flowsheet |
| pHUrine*^a^* | True | Numeric | - | Urine pH. | EMR – lab results flowsheet |
| AntibodyScreenSaline*^a^* | True | Categorical | - | Unique values = positive, negative. | EMR – lab results flowsheet |
| Appearance | True | Categorical | - | Urine appearance.  Unique values = bloody, clear, hazy, purulent, NotMeasured. | EMR – lab results flowsheet |
| Bacteria | True | Categorical | - | Bacteria in urine. Unique values = none, rare, few, moderate, many, NotMeasured. | EMR – lab results flowsheet |
| ColorUrine | True | Categorical | - | Urine color. Unique values = no color, yellow, orange, amber, bloody, other, NotMeasured. | EMR – lab results flowsheet |
| Crystals | True | Categorical | #/HPF*^d^* | Crystals in urine. Unique values = none, rare, few, moderate, many, NotMeasured. | EMR – lab results flowsheet |
| KetonesQualitative | True | Categorical | - | Ketones in urine. Unique values = negative, trace, small, moderate, large, NotMeasured. | EMR – lab results flowsheet |
| LeukocyteEsterase | True | Categorical | - | Leukocyte esterase in urine. Unique values = negative, trace, small, moderate, large, NotMeasured. | EMR – lab results flowsheet |
| Nitrite | True | Categorical | - | Nitrites in urine. Unique values = positive, negative, NotMeasured. | EMR – lab results flowsheet |
| ProteinSemiquantitative | True | Categorical | - | Unique values = negative, trace, 1+, 2+, NotMeasured. | EMR – lab results flowsheet |

*^a^* These variables have a corresponding missingness indicator variable whose names are expressed as the respective variable name appended with “_ind”.

*^b^* Transfusion rate (mL/kg/min) is calculated as the total volume of transfusion administered in 1 hour period divided by (patient weight (kg) x 60 (minutes))

*^c^* Transfusion volume since PICU admission is calculated as the total volume of transfusion (mL) administered since PICU admission.

*^d^* High power field

**Supplementary Table 2.** Patient characteristics by multiple organ dysfunction status based on Goldstein et al. and Proulx et al. criteria

| **Characteristic*^a^*** | **All PICU**  **encounters**  **(n = 2565)** | **Goldstein criteria** | | | **Proulx criteria** | | |
| --- | --- | --- | --- | --- | --- | --- | --- |
|  |  | **MOD**  **(n = 893)** | **No MOD**  **(n = 1672)** | ***p*- value***^b^* | **MOD**  **(n = 692)** | **No MOD**  **(n = 1873)** | ***p*- value**^†^ |
| LOS, days | 1.9 [1.0 - 4.0] | 4.6 [2.1 - 8.9] | 1.4 [0.9 - 2.3] | <0.001 | 5.0 [2.3 - 10.9] | 1.5 [0.9 - 2.6] | <0.001 |
| **Demographics** | | | | | | | |
| Age, years | 5.3 [1.4 - 12.4] | 4.0 [0.9 - 11.2] | 6.0 [1.8 - 12.9] | <0.001 | 2.2 [0.5 - 8.1] | 6.7 [2.3 - 13.2] | <0.001 |
| Males | 1405 (54.8) | 487 (54.5) | 918 (54.9) | 0.868 | 385 (55.6) | 1020 (54.5) | 0.623 |
| Weight, kg | 18.8 [10.0 - 40.0] | 15.7 [8.4 - 30.8] | 20.1 [11.1 - 44.7] | <0.001 | 12.3 [6.2 - 24.0] | 21.7 [12.0 - 44.0] | <0.001 |
| Race | | | | | | | |
| African American | 879 (34.3) | 279 (31.2) | 600 (35.9) | 0.018 | 242 (35.0) | 637 (34.0) | 0.673 |
| White | 1156 (45.1) | 416 (46.6) | 740 (44.3) | 0.261 | 299 (43.2) | 857 (45.8) | 0.264 |
| Other | 530 (20.7) | 198 (22.2) | 332 (19.9) | 0.167 | 151 (21.8) | 379 (20.2) | 0.380 |
| **Interventions** | | | | | | | |
| Respiratory support | | | | | | | |
| Mechanical ventilation | 610 (23.8) | 590 (66.1) | 20 (1.2) | <0.001 | 414 (59.8) | 196 (10.5) | <0.001 |
| Non-invasive positive pressure ventilation | 621 (24.2) | 382 (42.8) | 239 (14.3) | <0.001 | 307 (44.4) | 314 (16.8) | <0.001 |
| Supplemental oxygen without pressure | 1193 (46.5) | 610 (68.3) | 583 (34.9) | <0.001 | 457 (66.0) | 736 (39.3) | <0.001 |
| Room Air (no support) | 1763 (68.7) | 628 (70.3) | 1135 (67.9) | 0.211 | 531 (76.7) | 1232 (65.8) | <0.001 |
| Inhaled nitric oxide | 11 (0.4) | 7 (0.8) | 4 (0.2) | 0.057 | 8 (1.2) | 3 (0.2) | 0.002 |
| VIS*^c^* | 0.0 [0.0 - 0.0] | 0.0 [0.0 - 7.0] | 0.0 [0.0 - 0.0] | 0.171 | 0.0 [0.0 - 10.0] | 0.0 [0.0 - 0.0] | 0.100 |
| IS*^d^* | 0.0 [0.0 - 0.0] | 0.0 [0.0 - 2.0] | 0.0 [0.0 - 0.0] | 0.171 | 0.0 [0.0 - 7.0] | 0.0 [0.0 - 0.0] | 0.100 |
| ECMO | 25 (1.0) | 25 (2.8) | 0 (0.0) | <0.001 | 25 (3.6) | 0 (0.0) | <0.001 |
| Ventricular Assist Device | 29 (1.1) | 15 (1.7) | 14 (0.8) | 0.076 | 13 (1.9) | 16 (0.9) | 0.036 |
| Renal replacement therapy | 55 (2.1) | 39 (4.4) | 16 (1.0) | <0.001 | 38 (5.5) | 17 (0.9) | <0.001 |
| Plasmapheresis | 17 (0.7) | 17 (1.9) | 0 (0.0) | <0.001 | 16 (2.3) | 1 (0.1) | <0.001 |
| Transfusions | | | | | | | |
| RBC*^e^* | 417 (16.3) | 334 (37.4) | 83 (5.0) | <0.001 | 288 (41.6) | 129 (6.9) | <0.001 |
| Platelets | 127 (5.0) | 113 (12.7) | 14 (0.8) | <0.001 | 104 (15.0) | 23 (1.2) | <0.001 |
| Plasma | 106 (4.1) | 99 (11.1) | 7 (0.4) | <0.001 | 96 (13.9) | 10 (0.5) | <0.001 |
| Cryoprecipitate | 46 (1.8) | 45 (5.0) | 1 (0.1) | <0.001 | 45 (6.5) | 1 (0.1) | <0.001 |
| **Diagnostic categories** | | | | | | | |
| Operative cardiac | 164 (6.4) | 123 (13.8) | 41 (2.5) | <0.001 | 106 (15.3) | 58 (3.1) | <0.001 |
| Non-operative cardiac | 145 (5.7) | 46 (5.2) | 99 (5.9) | 0.473 | 39 (5.6) | 106 (5.7) | 1.000 |
| Neonatal respiratory | 42 (1.6) | 33 (3.7) | 9 (0.5) | <0.001 | 35 (5.1) | 7 (0.4) | <0.001 |
| Pediatric respiratory | 963 (37.5) | 475 (53.2) | 488 (29.2) | <0.001 | 341 (49.3) | 622 (33.2) | <0.001 |
| Other | 1251 (48.8) | 216 (24.2) | 1035 (61.9) | <0.001 | 171 (24.7%) | 1080 (57.7) | <0.001 |
| **Most common diagnoses** | | | | | | | |
| Seizures | 429 (16.7) | 236 (26.4) | 193 (11.5) | <0.001 | 137 (19.8) | 292 (15.6) | 0.012 |
| Sepsis | 347 (13.5) | 206 (23.1) | 141 (8.4) | <0.001 | 173 (25.0) | 174 (9.3) | <0.001 |
| Status asthmaticus | 208 (8.1) | 22 (2.5) | 186 (11.1) | <0.001 | 24 (3.5) | 184 (9.8) | <0.001 |
| Renal insufficiency | 171 (6.7) | 128 (14.3) | 43 (2.6) | <0.001 | 111 (16.0) | 60 (3.2) | <0.001 |
| Genetic abnormality or syndrome | 160 (6.2) | 78 (8.7) | 82 (4.9) | <0.001 | 53 (7.7) | 107 (5.7) | 0.080 |
| Scoliosis status post spinal fusion | 136 (5.3) | 48 (5.4) | 88 (5.3) | 0.926 | 16 (2.3) | 120 (6.4) | <0.001 |
| Pneumonia | 90 (3.5) | 71 (8.0) | 19 (1.1) | <0.001 | 69 (10.0) | 21 (1.1) | <0.001 |
| Traumatic brain injury | 69 (2.7) | 22 (2.5) | 47 (2.8) | 0.701 | 24 (3.5) | 45 (2.4) | 0.168 |
| Cardiopulmonary resuscitation | 66 (2.6) | 62 (6.9) | 4 (0.2) | <0.001 | 57 (8.2) | 9 (0.5) | <0.001 |
| Cerebral hemorrhage | 38 (1.5) | 21 (2.4) | 17 (1.0) | 0.010 | 16 (2.3) | 22 (1.2) | 0.042 |
| **PCCC categories*^f^*** | | | | | | | |
| Neurologic and Neuromuscular | 467 (18.2) | 216 (24.2) | 251 (15.0) | <0.001 | 131 (18.9) | 336 (17.9) | 0.565 |
| Cardiovascular | 165 (6.4) | 95 (10.6) | 70 (4.2) | <0.001 | 82 (11.8) | 83 (4.4) | <0.001 |
| Respiratory | 107 (4.2) | 61 (6.8) | 46 (2.8) | <0.001 | 43 (6.2) | 64 (3.4) | 0.003 |
| Renal and urologic | 114 (4.4) | 53 (5.9) | 61 (3.6) | 0.009 | 49 (7.1) | 65 (3.5) | <0.001 |
| Gastrointestinal | 49 (1.9) | 20 (2.2) | 29 (1.7) | 0.368 | 19 (2.7) | 30 (1.6) | 0.073 |
| Hematologic or immunologic | 99 (3.9) | 60 (6.7) | 39 (2.3) | <0.001 | 53 (7.7) | 46 (2.5) | <0.001 |
| Metabolic | 68 (2.7) | 27 (3.0) | 41 (2.5) | 0.439 | 23 (3.3) | 45 (2.4) | 0.213 |
| Other congenital or genetic defects | 102 (4.0) | 45 (5.0) | 57 (3.4) | 0.056 | 30 (4.3) | 72 (3.8) | 0.570 |
| Malignancy | 266 (10.4) | 105 (11.8) | 161 (9.6) | 0.103 | 81 (11.7) | 185 (9.9) | 0.189 |
| Premature and Neonatal | 0 (0) | 0 (0) | 0 (0) | - | 0 (0) | 0 (0) | - |

*^a^* Continuous variables are presented as medians [25^th^ - 75^th^ percentiles] and categorical variables as counts (frequencies in %).

*^b^ p*-values are computed using Wilcoxon rank sum test to test for similarity in distribution across MOD and no-MOD PICU encounters.

*^c^* Vasoactive inotropic score.

*^d^* Inotropic score.

*^e^* Red blood cells.

*^f^* Pediatric complex chronic conditions(7,8).

**Supplementary Table 3.** Distribution of high risk intervals (HRI) normalized by length of available data in positive predictions for all methods using both Goldstein and Proulx criteria

| **Method** | **HRI normalized by length of available data** | | |
| --- | --- | --- | --- |
|  | **True positives^a^** | **False positives^a^** | ***p*-value*^b^*** |
| **Goldstein criteria** | | | |
| Random Forest | 1.00 [1.00 - 1.00] | 0.21 [0.06 - 0.74] | < 0.001 |
| XGBoost | 1.00 [0.44 - 1.00] | 0.12 [0.02 - 0.24] | < 0.001 |
| GLMBoost | 1.00 [0.93 - 1.00] | 0.22 [0.10 - 0.63] | < 0.001 |
| LassoGLM | 1.00 [0.94 - 1.00] | 0.07 [0.03 - 0.48] | < 0.001 |
| **Proulx criteria** | | | |
| Random Forest | 1.00 [0.83 - 1.00] | 0.70 [0.19 - 0.90] | < 0.001 |
| XGBoost | 1.00 [0.66 - 1.00] | 0.35 [0.03 - 0.75] | < 0.001 |
| GLMBoost | 1.00 [0.70 - 1.00] | 0.42 [0.14 - 0.72] | < 0.001 |
| LassoGLM | 1.00 [0.40 - 1.00] | 0.36 [0.01 - 0.66] | < 0.001 |

^a^ Expressed as median [25^th^ - 75^th^ percentile]

***^b^*** *p*-value obtained from comparing HRI normalized by length of available data between true and false positives using Wilcoxon rank sum test.

**Supplementary Table 4.** Early warning times (EWT) relative to length of available data across high and moderate risk group vs. low risk group based on spectral clustering of risk-score trajectories of positive predictions for all methods using both Goldstein and Proulx criteria

| **Method** | **Early warning time relative to length of available data** | | |
| --- | --- | --- | --- |
|  | **High + moderate risk group^a^** | **Low risk group^a^** | ***p*-value*^b^*** |
| **Goldstein criteria** | | | |
| Random Forest | 0 [0 - 0] | 0 [0 - 0.04] | <0.001 |
| XGBoost | 0 [0 - 0] | 0.04 [0.02 - 0.13] | <0.001 |
| GLMBoost | 0 [0 - 0] | 0.07 [0 - 0.81] | <0.001 |
| LassoGLM | 0 [0 - 0] | 0 [0 - 0.03] | 0.166 |
| **Proulx criteria** | | | |
| Random Forest | 0 [0 - 0] | 0.21 [0.03 - 0.34] | <0.001 |
| XGBoost | 0 [0 - 0] | 0.21 [0 - 0.52] | <0.001 |
| GLMBoost | 0 [0 - 0] | 0.1 [0 - 0.32] | <0.001 |
| LassoGLM | 0 [0 - 0] | 0.14 [0 - 0.35] | <0.001 |

^a^ Expressed as median [25^th^ - 75^th^ percentile]

***^b^*** *p*-value obtained from comparing EWT relative to length of available data between high + moderate risk group and low-risk group using Wilcoxon rank sum test.

**Supplementary Table 5.** Positive predictive value across quartiles of risk-scores for positive predicted cases immediately following high-risk alert events

| **Risk-score percentiles** | **Positive predictive value** | | | |
| --- | --- | --- | --- | --- |
|  | **Random Forest** | **XGBoost** | **GLMBoost** | **LassoGLM** |
| **Goldstein criteria** | | | | |
| 0 – 25 | 0.29 | 0.47 | 0.35 | 0.40 |
| 25 – 50 | 0.44 | 0.57 | 0.59 | 0.60 |
| 50 – 75 | 0.94 | 0.93 | 0.88 | 0.73 |
| 75 – 100 | 1.00 | 0.93 | 1.00 | 1.00 |
| **Proulx criteria** | | | | |
| 0 – 25 | 0.69 | 0.71 | 0.41 | 0.59 |
| 25 – 50 | 0.57 | 0.57 | 0.66 | 0.72 |
| 50 – 75 | 1.00 | 0.96 | 0.97 | 0.93 |
| 75 – 100 | 1.00 | 1.00 | 1.00 | 1.00 |

**Supplementary Table 6.** Table of 20 most important features obtained from each of the four methods using Goldstein et al. organ dysfunction criteria

| Random Forest | | XGBoost | | GLMBoost | | LassoGLM | |
| --- | --- | --- | --- | --- | --- | --- | --- |
| Feature | Rel. Imp^a^ | Feature | Rel. Imp^a^ | Feature | Rel. Imp^a^ | Feature | Rel. Imp^a^ |
| aPTT | 100.0 | aPTT_ind | 100.0 | wbc | 100.0 | wbc | 100.0 |
| aPTT_ind | 79.6 | aPTT | 71.3 | GCS | 95.3 | GCS | 97.5 |
| PT | 67.5 | age | 59.0 | VIS | 90.3 | VIS | 77.5 |
| RBC_transfusion_vol | 64.7 | AspartateAminoTransferase | 57.3 | ColorUrineBloody | 89.0 | aPTT | 75.6 |
| PT_ind | 50.6 | weight | 48.8 | EosinophilNumber | 87.8 | EosinophilNumber | 75.5 |
| temp_diff | 44.3 | RBC_transfusion_vol | 47.8 | MonocytePercent | 80.0 | BUN | 71.7 |
| pH_art | 37.9 | S2F | 43.7 | BUN | 74.6 | ColorUrineBloody | 66.9 |
| BUN | 33.4 | ImmatureGranPercent | 34.6 | temp_diff | 56.2 | MonocytePercent | 56.9 |
| RBCDistributionWidth | 32.5 | BUN | 31.4 | PT | 55.9 | glucose | 40.8 |
| AspartateAminoTransferase | 32.5 | GCS | 31.3 | CalculatedBicarbArterial | 35.8 | ImmatureGranPercent | 39.0 |
| MonocytePercent | 32.4 | MonocytePercent | 28.4 | ImmatureGranPercent | 34.5 | OI_ind | 37.5 |
| CalculatedBicarbArterial | 32.3 | MeanCorpuscularVolume | 26.6 | aPTT | 32.6 | S2F_ind | 31.1 |
| MonocyteNumber | 29.1 | CalculatedBicarbArterial | 25.4 | temperature | 32.0 | temperature | 28.7 |
| CO2 | 25.7 | SpecimenpH | 20.2 | S2F_ind | 31.2 | temp_diff | 26.1 |
| AntibodyScreenSalineAHGNEGATIVE | 24.7 | AntibodyScreenSalineAHGNotMeasured | 19.2 | RBCCountUrine | 29.5 | CalculatedBicarbArterial | 25.8 |
| weight | 22.8 | RBCDistributionWidth | 18.4 | plt_transfusion_vol | 29.1 | weight | 24.6 |
| PaCO2 | 22.2 | AlanineAminoTransferase | 17.8 | glucose | 29.0 | EpithelialCells | 21.2 |
| age | 21.0 | temp_diff | 17.5 | OI_ind | 29.0 | CO2 | 19.6 |
| AntibodyScreenSalineAHGNotMeasured | 20.4 | Calcium | 15.0 | AlkalinePhosphatase | 21.0 | AntibodyScreenSalineAHGNEGATIVE | 17.4 |
| AlanineAminoTransferase | 19.8 | AntibodyScreenSalineAHGNEGATIVE | 14.7 | AbsoluteNeutrophilCount | 20.3 | temp_diff_ind | 16.0 |

^a^Rel Imp (relative importance score) obtained by normalizing the importance scores to have maximum value 100.

**Supplementary Table 7.** Table of 20 most important features obtained from each of the four methods using Proulx et al. organ dysfunction criteria.

| Random Forest | | XGBoost | | GLMBoost | | LassoGLM | |
| --- | --- | --- | --- | --- | --- | --- | --- |
| Feature | Rel. Imp^a^ | Feature | Rel. Imp^a^ | Feature | Rel. Imp^a^ | Feature | Rel. Imp^a^ |
| RBC_transfusion_vol | 100.0 | RBC_transfusion_vol | 100.0 | VIS | 100.0 | VIS | 100.0 |
| aPTT_ind | 72.2 | aPTT | 90.6 | Bilirubin | 19.3 | Bilirubin | 13.4 |
| PT | 50.4 | age | 57.5 | EosinophilPercent | 7.8 | AbsoluteLymphocyteCount | 5.8 |
| aPTT | 50.2 | S2F | 34.8 | BUN | 4.0 | EosinophilPercent | 5.8 |
| PT_ind | 41.5 | temp_diff_ind | 34.6 | age | 3.8 | wbc | 4.2 |
| age | 41.4 | weight | 31.6 | Creatinine | 3.1 | Creatinine | 4.0 |
| temp_diff | 33.4 | RBCDistributionWidth | 25.6 | peri_dialysis | 3.0 | RR | 3.5 |
| RBCDistributionWidth | 32.5 | aPTT_ind | 21.1 | aPTT | 2.7 | age | 3.4 |
| temp_diff_ind | 32.4 | AntibodyScreenSalineAHGNEGATIVE | 18.9 | CalculatedBicarbnonarterial | 2.7 | BUN | 2.8 |
| weight | 31.6 | AntibodyScreenSalineAHGNotMeasured | 18.3 | RR | 2.6 | PT | 2.7 |
| pH_art | 30.7 | CalculatedBicarbnonarterial | 18.0 | ImmatureGranPercent | 2.6 | Monocytes | 2.3 |
| AntibodyScreenSalineAHGNotMeasured | 30.4 | MeanCorpuscularVolume | 17.2 | AbsoluteLymphocyteCount | 2.6 | peri_dialysis | 2.2 |
| CalculatedBicarbArterial | 29.1 | MagnesiumSerum | 14.1 | AntibodyScreenSalineAHGNotMeasured | 1.9 | CalculatedBicarbnonarterial | 2.0 |
| CalculatedBicarbnonarterial | 27.3 | IonizedCalcium | 13.9 | IonizedCalcium | 1.9 | ImmatureGranPercent | 1.8 |
| AntibodyScreenSalineAHGNEGATIVE | 24.2 | CO2 | 13.1 | S2F_ind | 1.8 | temperature | 1.8 |
| CReactiveProtein_ind | 23.6 | pH_nonart | 12.5 | aPTT_ind | 1.7 | plt_transfusion_vol | 1.7 |
| S2F | 22.3 | SpecimenpH | 12.2 | CalculatedBicarbArterial | 1.7 | NBP_S | 1.6 |
| CReactiveProtein | 21.5 | AlanineAminoTransferase | 10.9 | GCS | 1.6 | CO2 | 1.6 |
| S2F_ind | 20.4 | CReactiveProtein_ind | 10.7 | temp_diff_ind | 1.5 | glucose | 1.6 |
| CO2 | 19.7 | BUN | 9.7 | glucose | 1.3 | S2F_ind | 1.6 |

^a^ Rel Imp (relative importance score) obtained by normalizing the importance scores to have maximum value 100.

**References**

1. Sharafoddini A, Dubin JA, Maslove DM, Lee J. A new insight into missing data in intensive care unit patient profiles: Observational study. J Med Internet Res. 2019 Jan 1;21(1).

2. Bembea MM, Nadkarni VM, Diener-West M, Venugopal V, Carey SM, Berg RA, et al. Temperature patterns in the early postresuscitation period after pediatric inhospital cardiac arrest. Pediatr Crit Care Med. 2010;11(6):723–30.

3. Gaies MG, Gurney JG, Yen AH, Napoli ML, Gajarski RJ, Ohye RG, et al. Vasoactive-inotropic score as a predictor of morbidity and mortality in infants after cardiopulmonary bypass. Pediatr Crit Care Med. 2010;11(2):234–8.

4. Jouvet P, Thomas NJ, Willson DF, Erickson S, Khemani R, Smith L, et al. Pediatric Acute Respiratory Distress Syndrome: Consensus Recommendations from the Pediatric Acute Lung Injury Consensus Conference. In: Pediatric Critical Care Medicine. Lippincott Williams and Wilkins; 2015. p. 428–39.

5. Thomas NJ, Shaffer ML, Willson DF, Shih MC, Curley MAQ. Defining acute lung disease in children with the oxygenation saturation index*. Pediatr Crit Care Med. 2010;11(1):12.

6. Khemani RG, Thomas NJ, Venkatachalam V, Scimeme JP, Berutti T, Schneider JB, et al. Comparison of SpO2 to PaO2 based markers of lung disease severity for children with acute lung injury*. Crit Care Med. 2012 Apr;40(4):1309–16.

7. Feudtner C, Feinstein JA, Zhong W, Hall M, Dai D. Pediatric complex chronic conditions classification system version 2: Updated for ICD-10 and complex medical technology dependence and transplantation. BMC Pediatr. 2014;14(1):1–7.

8. Feudtner C, Christakis DA, Connell FA, Study AP, State W. Pediatric Deaths Attributable to Complex Chronic Conditions : Pediatrics. 2000;106(1):205.
